# Heterogeneity and dynamics of active Kras-induced dysplastic lineages from mouse corpus stomach

**DOI:** 10.1038/s41467-019-13479-6

**Published:** 2019-12-05

**Authors:** Jimin Min, Paige N. Vega, Amy C. Engevik, Janice A. Williams, Qing Yang, Loraine M. Patterson, Alan J. Simmons, R. Jarrett Bliton, Joshua W. Betts, Ken S. Lau, Scott T. Magness, James R. Goldenring, Eunyoung Choi

**Affiliations:** 1 Department of Surgery, Vanderbilt University School of Medicine, Nashville, TN 37232 USA; 2 Epithelial Biology Center, Vanderbilt University School of Medicine, Nashville, TN 37232 USA; 3 Department of Cell and Developmental Biology, Vanderbilt University School of Medicine, Nashville, TN 37232 USA; 4 Cell Imaging Share Resource, Vanderbilt University School of Medicine, Nashville, TN 37232 USA; 5 Institute of Pathogen Biology, School of Basic Medical Sciences, Shandong University, Jinan, China; 6 Center for GI Biology and Disease, University of North Carolina at Chapel Hill, Chapel Hill, NC 27599 USA; 7 UNC Departments of Cell Biology and Physiology, University of North Carolina at Chapel Hill, Chapel Hill, NC 27599 USA; 8 University of North Carolina Chapel Hill/ North Carolina State University joint Departments of Biomedical Engineering, Chapel Hill, NC 27599 USA; 9 Department of Medicine, University of North Carolina at Chapel Hill, Chapel Hill, NC 27599 USA; 10 Nashville VA Medical Center, Nashville, TN 37232 USA

**Keywords:** Cancer models, Cancer stem cells, Oncogenes

## Abstract

Dysplasia is considered a key transition state between pre-cancer and cancer in gastric carcinogenesis. However, the cellular or phenotypic heterogeneity and mechanisms of dysplasia progression have not been elucidated. We have established metaplastic and dysplastic organoid lines, derived from Mist1-Kras(G12D) mouse stomach corpus and studied distinct cellular behaviors and characteristics of metaplastic and dysplastic organoids. We also examined functional roles for Kras activation in dysplasia progression using Selumetinib, a MEK inhibitor, which is a downstream mediator of Kras signaling. Here, we report that dysplastic organoids die or show altered cellular behaviors and diminished aggressive behavior in response to MEK inhibition. However, the organoids surviving after MEK inhibition maintain cellular heterogeneity. Two dysplastic stem cell (DSC) populations are also identified in dysplastic cells, which exhibited different clonogenic potentials. Therefore, Kras activation controls cellular dynamics and progression to dysplasia, and DSCs might contribute to cellular heterogeneity in dysplastic cell lineages.

## Abstract (web summary)

How a precancerous form (dysplasia) becomes gastric cancer is unclear. Here, the authors assess the role of Kras activation in heterogenous dysplastic cells in murine stomach corpus organoids, identifying two dysplastic stem cell populations and show that MEK inhibition causes alterations in cell behavior.

## Introduction

Intestinal type-gastric cancer, the most common type of gastric cancer^1,2^, develops within a field of metaplastic mucosal lineages. Neoplasia in the stomach corpus represents the most prominent example of cancer developing through a cascade of metaplastic, dysplastic and neoplastic changes^1,3,4^. Previous studies by our group and others using a number of different mouse models have identified the initiating steps of gastric carcinogenesis, such as spasmolytic polypeptide-expressing metaplasia (SPEM) development and its progression to intestinal metaplasia (IM)^5–8^. Although SPEM and IM are considered as central to the development of gastric cancer^9^, it is still unclear whether these metaplasias have a capacity to evolve directly into neoplasia. Furthermore, the identity of dominant signaling pathways or master regulators promoting metaplasia progression to neoplasia remain unclear, in part because no mouse models to date recapitulate the full spectrum of gastric carcinogenesis

A tumor in many types of cancers, including gastric cancer, is composed of many different cell populations, including cancer cells, immune cells, mesenchymal cells and endothelial cells. Cancer cells themselves have heterogenous intra-tumor populations^10,11^. This cellular heterogeneity can be caused by altered gene activation with or without genetic mutations in individual cells^12,13^. Upregulation of Kras activity, a key signaling pathway contributing to gastric cancer development and progression, has been observed in up to 40% of human intestinal type gastric cancers^14–16^. Recently our investigations using a mouse model, which induces active Kras expression in Mist1-expressing chief cells in the stomach corpus mucosa, showed that metaplasia progression is controlled by Kras activity and reversed by an inhibitor of MEK, a downstream mediator of the Kras signaling pathway^5,17^. However, the cellular mechanisms or biological functions of Kras activation on cellular heterogeneity during metaplasia transition to neoplasia remain obscure.

Cancer stem cells are considered a central source of the diverse cell populations in cancer and have essential roles in cancer cell differentiation, tumor growth, recurrence and even cancer metastasis and therapeutic treatment resistance^18,19^. Therefore, intra-tumor heterogeneity has become a crucial factor in determining successful therapeutic treatments. In gastric cancer, cellular heterogeneity is commonly observed and a variety of putative markers for cancer stem cells are present in human gastric cancer tissues^20–22^. However, the cellular heterogeneity and the presence of stem cells in dysplasia as a precursor of gastric cancer have not been explored. Thus, the cellular heterogeneity and plasticity within the dysplastic niche contributing to gastric cancer initiation and progression are largely unknown.

In this study, we report the development of in vitro gastric metaplastic and dysplastic organoid models to investigate the cellular characteristics and behaviors of metaplasia and dysplasia in the stomach corpus. These organoid lines recapitulate the phenotypes and characteristics observed in the gastric carcinogenesis cascade, as observed in activated Kras-induced mouse models and in human gastric cancer. We also define the cellular heterogeneity and distinct phenotypes of dysplastic cells and identified two different stem cell populations in dysplasia.

## Results

### Establishment of metaplastic or dysplastic organoids

**Fig. 1:**
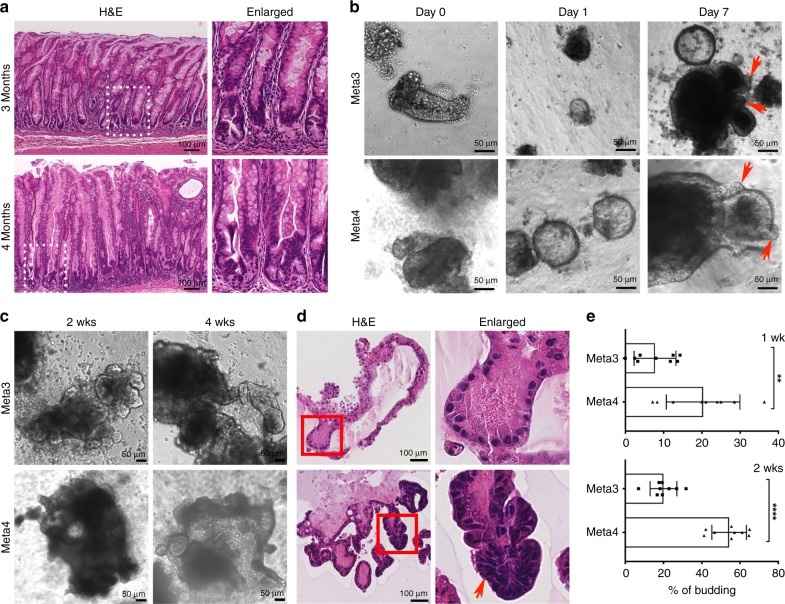
Establishment and characterization of Meta3 and Meta4 organoid lines. **a** H&E stained slides show stomach mucosa used to isolate metaplastic or dysplastic glands only from the corpus area of Mist1-Kras mouse stomachs at 3 or 4 (Meta3 or Meta4) months after tamoxifen injection. Dotted boxes indicate enlarged area. Scale bars indicate 100 μm. **b** Phase contrast images of Meta3 and Meta4 organoids captured 0, 1, and 7 days after plating in Matrigel. Red arrows indicate budding structures. Scale bars indicate 50 μm. **c** Phase contrast images of Meta3 and Meta4 captured at 2 or 4 weeks in 3D culture. Scale bars indicate 50 μm. **d** Paraffin embedded sections from Meta3 or Meta4 organoids at 4 weeks in 3D culture examined by H&E staining. Red boxes indicate enlarged area. Red arrow indicates a representative multilayered area of Meta4. Scale bars indicate 100 μm. **e** Quantitation of the average budding rate in Meta3 and Meta4 at 1 or 2 weeks in 3D cultures. Source data are provided as a Source Data file. Data are presented as mean values with standard deviation (*n* = 9). *P*-values were calculated using unpaired two-tailed *t*-test. **P = 0.004; ****P < 0.0001.

To study the cellular characteristics and behaviors of metaplasia and dysplasia, we first established two different organoid lines derived from Mist1-Kras mouse stomach corpus mucosa. As we have previously reported^5^, H&E staining confirmed the development of metaplasia at 3 months after tamoxifen injection and the presence of glands with dysplastic cells at 4 months after tamoxifen injection (Fig. 1a). We isolated glands in gastric tissues from the Mist1-Kras stomach corpus at 3 or 4 months after tamoxifen injection and plated them in Matrigel for 3-dimensional (3D) culture. The isolated glands formed spherical structures one day after plating in Matrigel (Fig. 1b). All of the organoid lines have undergone continuous passaging following derivation for over 1 year and form distinguishable structures in 3D cultures (Supplementary Fig. 1B, C).

We assessed growth and phenotypic changes in the organoids. While gastroids derived from normal stomach corpus mucosa form only spheroid structures^23,24^ (Supplementary Fig. 1D), the Meta3 and Meta4 organoids displayed more dynamic behaviors and complex structures. Both Meta3 and Meta4 organoids formed complicated budding or cyst-like structures with patches of Ki67-positive cells between 2 and 4 weeks in 3D culture (Fig. 1c and Supplementary Fig. 1E). Each organoid line generated from three independent mouse tissue samples showed complex structures with budding formation at 2 weeks in culture (Supplementary Fig. 1A). The budding formation in Meta4 was significantly increased at both 1 and 2 weeks in culture, compared to Meta3 organoids, and about 50% of Meta4 organoids showed budding structures at 2 weeks in culture (Fig. 1e). The Meta3 organoids developed large budding structures with a single monolayer of cells throughout most of the organoids within budding extensions (Fig. 1d). In contrast, the Meta4 cells displayed more disorganized organoid structures with multiple layering of cells, common cytological characteristics of dysplastic epithelial cells (Fig. 1d, red arrow). This observation is consistent with the phenotypes displayed in thicker glands along with glandular fission at the gland bases in vivo in Mist1-Kras mouse corpus at 4 months after tamoxifen injection (Fig. 1a). We additionally examined the histological phenotypes of Meta3 and Meta4 organoids. Both Meta3 and Meta4 organoids expressed CD44v9 and Sox9 (Supplementary Fig. 2A). Also, expression of Cdx1, which is considered a marker for IM, was occasionally observed in both Meta3 and Meta4 organoids (Supplementary Fig. 2A, white arrows). In particular, Meta4 organoids continuously expressed Cortactin, which has critical roles for cancer cell invasion and migration^25–28^, through many rounds of passaging (Supplementary Fig. 2B). Thus, these organoid lines displayed unique characteristics of metaplastic lineages with Meta3 cells reflecting IM and Meta4 cells showing more dysplastic phenotypes.

### Distinct characteristics of Meta3 and Meta4 organoids

**Fig. 2:**
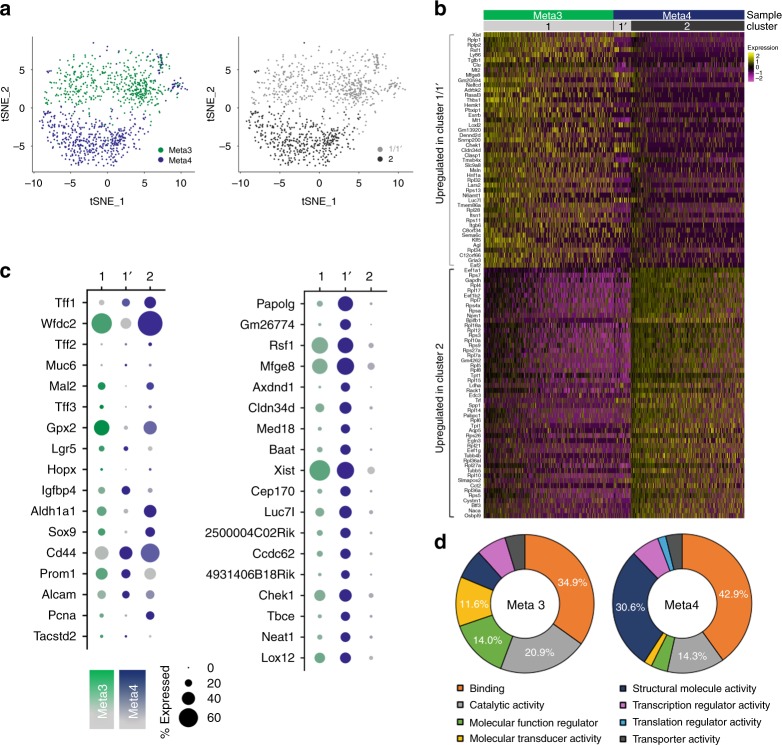
Single-cell RNA sequencing analysis of Meta3 and Meta4 cells. **a** t-SNE plot with overlay of Meta3 and Meta4 samples (left) and clustering of Meta3 and Meta4 datasets into subpopulations 1, 1’, and 2 (right). **b** Heatmap of the top 50 (approximately) upregulated genes found by differential expression analysis between subpopulations 1/1’ and 2. Upregulated genes were defined as those expressed in at least 25% of the cells in the sample with at least 0.1 log fold-change over the other subpopulation. *P*-values were calculated using a two-tailed the Wilcoxon Rank Sum test with Bonferroni correction and were <0.05. Rows correspond to individual genes and columns are individual cells, arranged by sample and subpopulation. Yellow corresponds to high expression, black corresponds to neither high nor low expression, and purple corresponds to low expression. **c** Dot plots showing selected markers for GCSCs, SPEM, IM, proliferation, and normal gastric epithelium, displayed by sample and subpopulation (left). Dot plots of upregulated genes identified by differential expression analysis of subpopulations 1’ and 2 within only the Meta4 sample, displayed by subpopulation and sample, including both Meta3 and Meta4 (right). The dot size represents the percent of cells within the subpopulation with detected expression of the gene and color intensity reflects the average expression in those cells with detectable gene expression. **d** PANTHER gene ontology classification results using the top 50 upregulated genes from differential expression analysis between Meta3 and Meta4 samples.

We used inDrop single-cell RNA-sequencing (scRNA-seq)^29–32^, and the Seurat pipeline^33,34^ to characterize further the Meta3 and Meta4 organoid lines as metaplastic or dysplastic, respectively (Supplementary Fig. 3A). Dimension reduction by PCA and visualization with t-Distributed Stochastic Neighbor Embedding (t-SNE)^35^ showed that the Meta3 and Meta4 samples separated almost entirely, suggesting major transcriptomic differences between the two organoid lines (Fig. 2a and Supplementary Fig. 3B, C). Unsupervised clustering and differential expression analyses revealed that the Meta3 sample has low heterogeneity and no subpopulations (cluster 1), while the Meta4 sample consists of a small, Meta3-like subpopulation (metaplastic, cluster 1’) and a dominant, Meta4-specific subpopulation (cluster 2) (Fig. 2a, Supplementary Figs. 4A-F and 5A, C). Genes upregulated in cluster 1’ cells were also upregulated in the Meta3 sample (Fig. 2a-c and Supplementary Fig. 5A, C). Differential expression analysis between clusters 1/1’ and 2 revealed several upregulated genes that support the metaplastic and dysplastic characterization of the organoids (Fig. 2b). Both Meta3 and Meta4 cells have high expression of many key markers of metaplasia, such as *Wfdc2*, *Mal2*, and *Gpx2*, and cancer stem cell (CSC) marker genes, such as *Cd44*, CD133 (*Prom1)*, and CD166 (*Alcam)* (Fig. 2c). Several differentially expressed genes between Meta3 and Meta4 were validated by qPCR (Supplementary Fig. 5B). PANTHER gene ontology analysis^36^ using upregulated genes for Meta3 and Meta4 samples (Supplementary Data 1) revealed upregulation of structural molecule activity and translation regulator activity in the Meta4 sample compared to the Meta3 sample (Fig. 2d). Taken together, the transcriptomic profiles of Meta3 and Meta4 samples are distinct and confirmed the cellular characteristics of Meta3 and Meta4 organoids as metaplastic or dysplastic organoids.

### Implanted Meta4 organoids engrafted in mouse stomachs

**Fig. 3:**
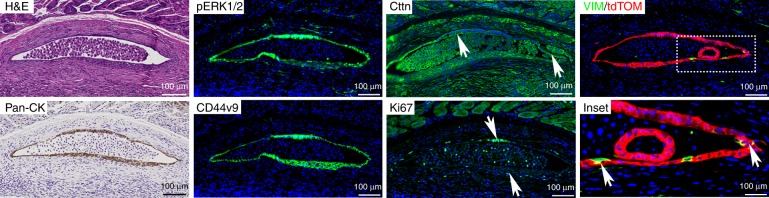
Orthotopic implantation of Meta4 organoids in mice. H&E stained slides show engrafted area in the stomach muscularis layer at 1 month after implantation. Immunostaining for an epithelial cell marker, pan-cytokeratin (pan-CK), a marker for Kras signaling downstream, phospho-ERK1/2 (pERK1/2), an EMT marker, Cortactin (CTTN), a metaplasia and gastric cancer marker, CD44v9, a proliferation marker, Ki67 and the red fluorescent protein tdTomato (tdTOM, red) with Vimentin (VIM, green). Dotted box indicates enlarged inset area. Scale bars indicate 100 μm. Source data are provided as a Source Data file.

To assess whether the dysplastic cells can survive and grow in vivo, we performed an orthotopic implantation study using Meta4 organoids. We implanted tdTOM-positive Meta4 organoids into the anterior stomach wall of C57BL/6 wildtype mice and sacrificed mice at 1 month after implantation. The implantation resulted in 40% local engraftment of Meta4 organoids within 1 month. The engraftments were mostly cystic and we did not observe gross tumors derived from Meta4 after implantation surgery. It is important to note that we also implanted Meta3 organoids into the stomach wall of C57BL/6 wildtype mice, however, no engraftment of Meta3 organoids was observed at 1 month after the implantation. A representative H&E stained image of engraftment demonstrates that Meta4 engrafted in the muscular layer of stomach and immune cells were infiltrating around the engraftment (Fig. 3). The engrafted cells were positive for tdTOM confirming that the origin of engraftment was Meta4 organoids and staining for phosphoERK1/2 and pan-cytokeratin, an epithelial cell marker, indicated that the engrafted cells were epithelial cells with active Kras signaling. The engrafted cells were positive for CD44v9 and Ki67, demonstrating that the engrafted cells maintained the characteristics of Meta4 organoids and were proliferative. In addition, we stained for Vimentin (VIM), which is a marker for epithelial-mesenchymal transition in cancer cells, and cortactin. We observed many apically oriented Cortactin-expressing engrafted cells, while only a few cells showed VIM immunoactivity (Fig. 3, white arrows). Therefore, this result shows that dysplastic cells, but not metaplastic cells, can survive and propagate in vivo.

### Cellular behaviors of Meta4 can be altered by MEK inhibition

**Fig. 4:**
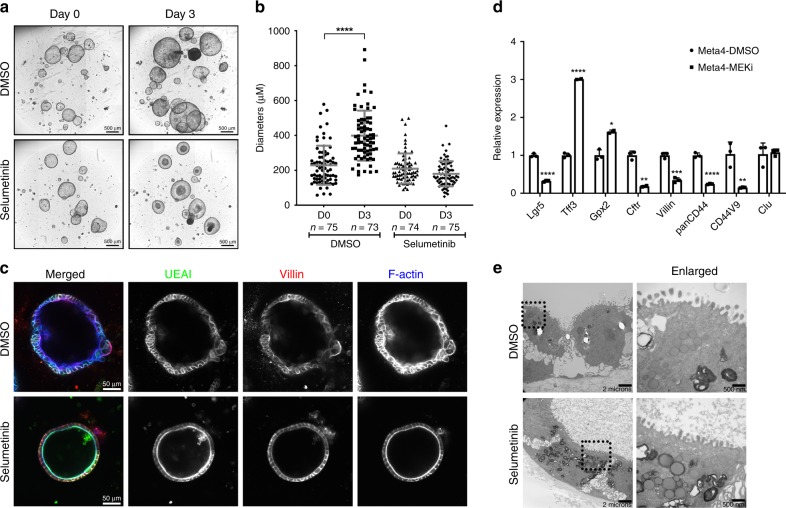
Examination of cellular changes in Meta4 organoids after MEK inhibition. **a** Meta4 organoids were treated with either DMSO containing control media or Selumetinib (1 μM) containing media for 3 days. Phase contrast images were captured before and 3 days after the DMSO vehicle or Selumetinib treatment. Scale bars indicate 500 μm. **b** Diameters of Meta4 organoids were manually measured before and after either DMSO vehicle or Selumetinib treatment. Data are presented as mean values with standard deviation. *P*-values were calculated using unpaired two-tailed *t*-test. ****P < 0.0001. **c** Co-immunostaining for markers of enterocyte apical membrane, UEAI, Villin and F-actin in paraffin sections of Meta4 treated with either DMSO vehicle or Selumetinib. Scale bars indicate 50 μm. **d** Expression of intestinal lineage marker transcripts after Selumetinib treatment. Quantitative PCR showing relative expression of intestinal lineage marker genes (*Lgr5, Lys, Tff3, Cdx1, Cdx2, Gpx2, Ctfr, Villin*, and *Muc2*), a CSC marker (pan*CD44*) and metaplasia markers (*CD44v9* and *Clu*) 3 days after Selumetinib treatment. *Lys, Cdx1, Cdx2*, and *Muc2* were not detected. Data are presented as mean values with standard deviation (*n* = 3). *P*-values were calculated using unpaired two-tailed *t*-test. *P < 0.05 (0.01; Gpx2), **P < 0.005 (0.002; Cftr, 0.009; CD44v9), ***P < 0.005 (0.0003; Villin), ****P < 0.0001 (Lgr5, Tff3, panCD44). **e** Transmission electron micrographs (TEM) of Meta4 organoids treated with either DMSO vehicle (above) or Selumetinib (below) for 3 days. Dotted boxes indicate enlarged area. Scale bars indicate 2 microns or 500 nm. Source data are provided as a Source Data file.

To examine whether the inhibition of the Kras signaling pathway can alter changes in cellular structures or behaviors and cell survival, we treated the Meta4 organoids with Selumetinib, a MEK inhibitor, at one or two days after passaging when the organoids show spheroidal structures. The inhibition of the Kras signaling pathway by Selumetinib was confirmed by decreased expression of phospho-Erk1/2 in three different Meta4 lines, while total Erk1/2 protein expression after the Selumetinib treatment was not changed (Supplementary Fig. 6A, B). Most Meta4 organoids treated with Selumetinib for 3 days showed decreased viability and the dead cells were extruded into the organoid lumen (Fig. 4a and Supplementary Fig. 6C and Supplementary Movie 1). However, some Meta4 organoids showed an ability to survive and retained spherical structures despite the Selumetinib treatment for 3 days, although they did not show an increase in size and showed decreases in *Ki67* gene expression level and Ki67-positive cells (Fig. 4a, b and Supplementary Fig. 6E, F). The Selumetinib-treated Meta4 organoids showed a thin epithelial layer and formed rounded spheroidal shapes, whereas the DMSO vehicle-treated organoids showed a thicker epithelial layer and irregular spheroidal shapes (Fig. 4c). We next stained Meta4 organoids with antibodies against intestinal enterocyte apical membrane markers, including UEAI, Villin and F-actin to examine the structural changes in treated cells. While the Meta4 organoids treated with DMSO vehicle did not show apical brush border staining, F-actin, Villin and UEAI strongly stained the apical membranes of Meta4 cells after Selumetinib treatment (Fig. 4c). Finally, the remaining Meta4 organoids after MEK inhibition did not survive after three passages, indicating that the Meta4 organoids do not sustain prolonged growth under MEK inhibition condition (Supplementary Fig. 6D).

These findings suggested that the Meta4 cells transitioned into a more differentiated state towards an absorptive intestinal enterocyte phenotype. qPCR with primers for intestinal lineage marker genes confirmed altered differentiation of the Meta4 organoids treated with Selumetinib, with increased the expression of *Tff3* and *Gpx2*, but expression of the intestinal stem cell marker, *Lgr5* was decreased (Fig. 4d).

Transmission electron micrographs of the Meta4 organoids treated with either DMSO vehicle or Selumetinib also showed remarkable differences and some similarities. The Meta4 cells treated with DMSO vehicle showed less complete polarization with a lack of clear lateral cell–cell contacts or basal surface attachment. Although both organoids displayed features of polarity, as they clearly showed microvilli on the apical surface, the Meta4 organoids treated with DMSO vehicle showed signs of piling and an increase in electron dense material (Fig. 4e). In contrast, the Selumetinib-treated cells showed luminal content and a larger compartment of cytoplasmic vesicles similar to the early stages of autophagy (Fig. 4e). Taken together, the data suggest that the Selumetinib-treated Meta4 cells are differentiating into an absorptive cell phenotype after MEK inhibition.

We additionally examined whether the Meta3 organoids showed these dynamic changes after MEK inhibition. The Meta3 organoids treated with Selumetinib for 3 days also did not grow in size (Supplementary Fig. 7A, B). While UEAI and Villin were not present in the DMSO vehicle-treated Meta3, the apical membrane markers were strongly expressed in Selumetinib-treated Meta3 (Supplementary Fig. 7C), and enterocyte lineage marker genes, such as *Tff3* and *Gpx2*, as well as *Lys* (Lysozyme) and *Cdx1* were also increased (Supplementary Fig. 7D). Furthermore, the immunostaining for Villin in the stomachs of Mist1-Kras mice treated with Selumetinib for 2 weeks at 3 months after tamoxifen injection confirmed an increase in Villin expression in the apical membrane of remaining metaplastic cells after the Selumetinib treatment (Supplementary Fig. 7E). These results suggest that MEK inhibition in both Meta3 and Meta4 cells inhibits growth and promotes the differentiation of cells into enterocyte-like lineages.

### Dysplastic behaviors are altered by Cortactin localization

**Fig. 5:**
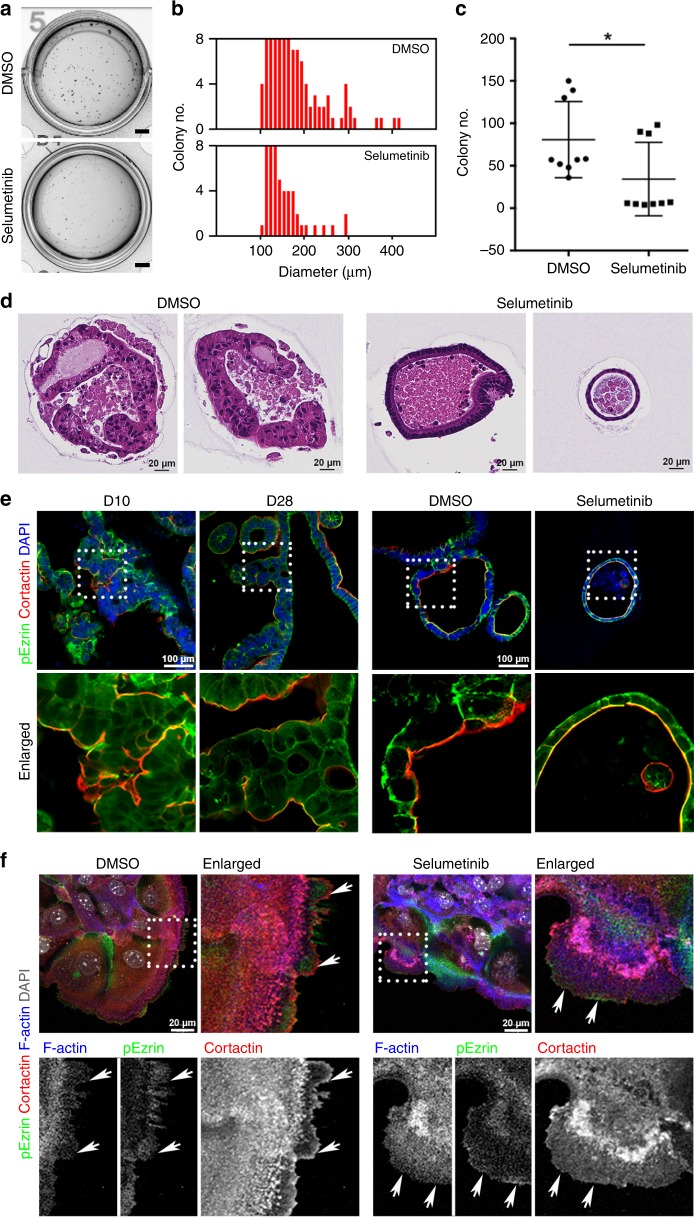
Inhibition of dysplastic behaviors of Meta4 organoids by MEK inhibition. **a**–**d** Inhibitory effect of Selumetinib treatment in soft agar culture for 2 weeks. **a** Phase contrast images show that colony formation was diminished by the treatment with Selumetinib (1 μM). Scale bars indicate 2 mm. **b** Colony diameter histogram shows the distribution of colony sizes. Mean value of diameters; DMSO vehicle treated Meta4 = 150.13 ± 47.2 μm and Selumetinib treated Meta4 = 133.13 ± 41.8 μm. **c** Graph shows a decrease in the number of colonies by Selumetinib treatment. Colonies over 100 μm in size were counted using a Gelcounter. Data are presented as mean values with standard deviation (*n* = 9). *P*-values were calculated using two-tailed Welch’s test. *P = 0.04. **d** Paraffin embedded sections of Meta4 organoids treated with either DMSO vehicle or Selumetinib at 2 weeks after in soft agar culture were examined by H&E staining. Scale bars indicate 20 μm. **e** Immunostaining for Cortactin (red) and an apical membrane marker, phospho-Ezrin (pEzrin, green) in Meta4 organoids at 10 or 28 days after 3D culture in Matrigel, or Meta4 treated with either DMSO vehicle or Selumetinib (1 μM) for 3 days. Nuclei were counterstained with DAPI (blue). Dotted boxes indicate enlarged area. Scale bars indicate 100 μm. **f** Immunostaining for Cortactin (red) and apical membrane markers, F-actin (blue) and pEzrin (green) in 2D monolayer cultured Meta4 treated with either DMSO vehicle or Selumetinib (1 μM) for 3 days. Nuclei were counterstained with DAPI (gray). Arrows indicate lamellipodia and dotted boxes indicate enlarged area. Scale bars indicate 20 μm. Source data are provided as a Source Data file.

Since MEK inhibition promoted apical differentiation and altered the morphology of Meta4 organoids, we next performed soft agar colony formation assays as a measure of anchorage independent growth to examine whether the Meta4 cells can be controlled by MEK inhibition in vitro. The Meta4 organoids treated with DMSO vehicle survived and grew larger in soft agar. However, many Meta4 organoids treated with Selumetinib failed to survive or showed arrested growth within 3 days of Selumetinib treatment (Supplementary Movie 2) and fewer colonies survived at 2 weeks in the Selumetinib-treated Meta4 compared to DMSO-treated Meta4 (Fig. 5a). While the Meta4 cells treated with DMSO vehicle formed higher numbers of colonies with a size of at least 100 μm diameter (80.77 ± 14.98) and grew into larger colonies (179 ± 8.37 μm), the colony number for Selumetinib-treated Meta4 was significantly decreased (34.43 ± 14.44), and the average size of colonies was slightly smaller (159 ± 13.98 μm) than DMSO vehicle-treated colonies (Fig. 5b, c). In addition, histological examination of surviving colonies in DMSO vehicle-treated wells displayed very disorganized dysplastic cell phenotypes similar to those we have observed in a long-term 3D culture of Meta4 organoids in Matrigel. In contrast, the surviving colonies in Selumetinib-treated wells showed rounded morphologies consisting of a monolayer of cells with very well-aligned and basally located nuclei, even in organoids that had grown to substantial sizes (Fig. 5d). These data suggest that the MEK inhibition alters the growth behavior of Meta4 organoids.

To examine an aggressive characteristic of the Meta4 organoids, we next examined the changes of Cortactin protein in Meta4 organoids after MEK inhibition. In 3D culture, Cortactin protein was observed in the apical membrane area of both DMSO vehicle-treated and Selumetinib-treated Meta4 organoids (Fig. 5e). In 2D monolayer culture, Cortactin was generally observed in the cytoplasm. However, in DMSO vehicle-treated Meta4 cells, Cortactin was also observed at the lamellipodia of cells at the leading edge of cell migration (Fig. 5f, white arrows). In contrast, the Cortactin expression at the lamellipodia was dramatically reduced after Selumetinib treatment and accumulated within the peri-nuclear region of the cells. At the same time, the expression of phospho-Ezrin, which is an apical brush border membrane marker in intestinal epithelial cells, was increased at the lamellipodia in Meta4 cells surviving Selumetinib treatment (Fig. 5f, white arrows). These data support the loss of aggressive phenotypes in Meta4 cells after MEK inhibition.

### Molecular changes in Meta4 cells after MEK inhibition

**Fig. 6:**
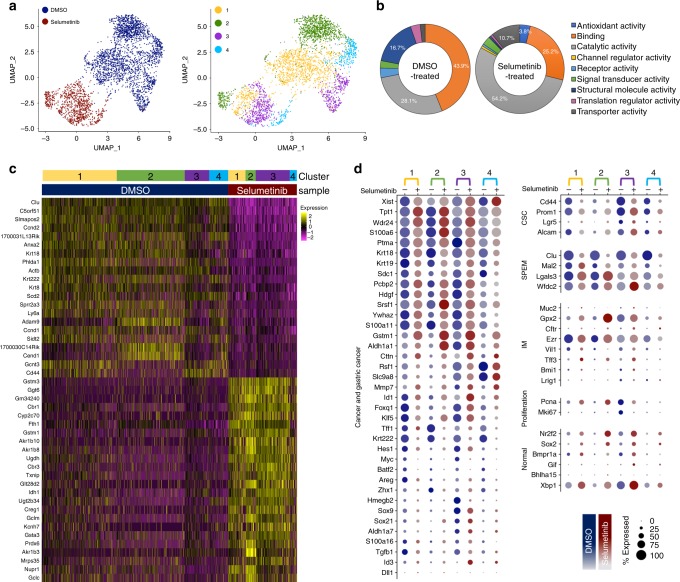
scRNA-seq analysis of Meta4 cells treated with either DMSO or Selumetinib. **a** UMAP of DMSO-treated and Selumetinib-treated Meta4 samples with sample overlay (left) and subpopulation-matched cluster overlay (right). **b** PANTHER gene ontology classification results from DMSO vehicle-treated and Selumetinib-treated Meta4 samples. Top 300 differentially expressed genes in either DMSO vehicle-treated or Selumetinib-treated Meta4 samples were used for the analysis. **c** Heatmap of upregulated genes in DMSO vehicle-treated or Selumetinib-treated samples. Upregulated genes were defined as those expressed in at least 50% of the cells in the sample with at least 0.75 log fold-change over the other sample. *P*-values were calculated using a two-tailed Wilcoxon Rank Sum test with Bonferroni correction and were <1e^−40^. Rows correspond to individual genes and columns are individual cells, arranged by sample and subpopulation. Yellow corresponds to high expression, black corresponds to neither high nor low expression, and purple corresponds to low expression. **d** Dot plots showing selected markers for cancer and gastric cancer (left), as well as markers for gastric cancer stem cells (GCSCs), SPEM, IM, proliferation, and normal gastric epithelium (right). Results are shown for matched subpopulations (1–4) and split by treatment (DMSO vehicle or Selumetinib). The dot size represents the percent of cells within the matched subpopulation with detected expression of the gene and color intensity reflects the average expression in those cells with detectable gene expression.

To evaluate transcriptomic changes and to define altered subpopulations or cellular heterogeneity of Meta4 after MEK inhibition, we performed scRNA-seq in Meta4 cells treated with either DMSO vehicle only or Selumetinib for 3 days^29–32^. We analyzed 2471 DMSO vehicle-treated Meta4 cells and 905 Selumetinib-treated Meta4 cells using the Seurat pipeline and our own subpopulation-matching algorithm^33,34^ (Supplementary Fig. 8A-C). Dimension reduction by PCA and visualization with Uniform Manifold Approximation and Projection (UMAP)^37^ showed that the DMSO vehicle-treated and Selumetinib-treated Meta4 samples separated almost entirely, suggesting major transcriptomic differences among DMSO vehicle-treated and Selumetinib-treated cells (Fig. 6a, Supplementary Fig. 8D-F). Gene ontology analysis using differentially expressed genes between the two samples (Supplementary Data 1) and the PANTHER classification system^36^ also supported the distinct gene expression profiles (Fig. 6b, c). DMSO vehicle-treated Meta4 cells exhibited upregulated structural molecule activity and binding related genes, such as *Krt7*, *Krt8*, and *Krt18*, which are also diagnostic markers in gastric cancer pathology^38^ (Fig. 6b-d, Supplementary Fig. 12A). Selumetinib-treated cells exhibited upregulation of catalytic and transporter activity, such as *Gpx2*, *Gclm*, *Gclc*, and *Akr1b3* and downregulation in the expression of stress markers, most notably *Clu* (Fig. 6b-d, Supplementary Fig. 12A).

**Fig. 7:**
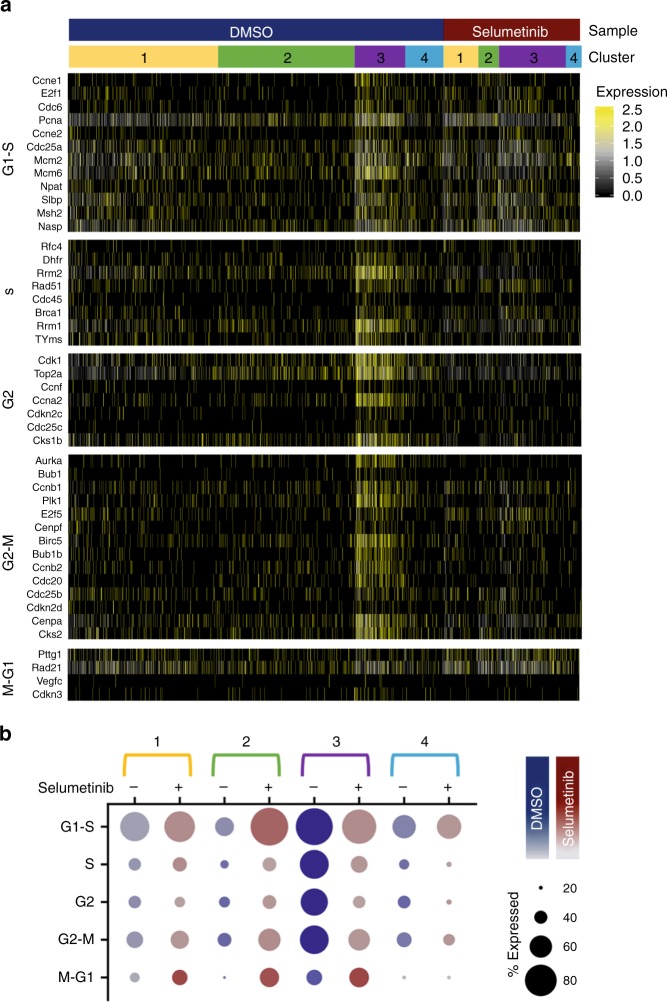
Cell cycle state in Meta4 cells treated with either DMSO or Selumetinib. **a** Heatmap of cell cycle genes categorized by phase in DMSO vehicle-treated and Selumetinib-treated Meta4 samples. Rows correspond to individual genes and columns are individual cells, arranged by sample and matched subpopulations. Yellow corresponds to high expression, gray corresponds to neither high nor low expression, and black corresponds to low expression. **b** Dot plots showing cell cycle meta-genes for each cell cycle phase. Results are shown for matched subpopulations (1–4) and split by treatment (DMSO vehicle or Selumetinib). The dot size represents the percent of cells within the matched subpopulation with detected expression of the gene and color intensity reflects the average expression in those cells with detectable gene expression.

To identify subpopulations of cells within DMSO vehicle-treated and Selumetinib-treated Meta4 samples, we performed unsupervised clustering analysis on each dataset (Supplementary Figs. 8G and 9A-H). Since the subpopulations within these samples were not aligned and could not be directly compared due to the perturbation of Selumetinib treatment, we performed a subpopulation-matching analysis that matches similar cell types between two samples using gene signatures (Supplementary Fig. 10A-C). This subpopulation-matching method identified four cell subpopulations present in both DMSO-vehicle and Selumetinib treatment groups, but the subpopulations differed in proportion in the samples (Fig. 6a, Supplementary Fig. 11B). Differential expression analysis identified many genes unique to each subpopulation in the context of DMSO-vehicle or Selumetinib treatment (Supplementary Fig. 11A). We next analyzed the characteristics of subpopulations using known markers of cancer, CSCs, SPEM, IM, proliferation, and normal gastric cells between matched subpopulations across the Selumetinib treatment (Fig. 6d). To investigate further the proliferative features in each subpopulation, we assessed the expression levels of genes specific to cell cycle stages^39^. This analysis revealed a strong proliferative signature in subpopulation 3 in the DMSO vehicle-treated sample (Fig. 7a, b), and the subpopulation 3 also expressed high levels of the proliferation markers, *Pcna* and *Mki67* (Fig. 6d). In addition to high expression of cell cycle and proliferation genes, subpopulation 3 highly expressed markers of CSCs and cancer-related genes, compared to the other three subpopulations (Fig. 6d). Although subpopulation 4 did not exhibit proliferative characteristics, there was high expression of many genes related to CSC or cancer. In addition, the data also showed a moderate increase in genes for normal gastric cells after MEK inhibition.

To investigate further the transcriptional activity of the subpopulations across Selumetinib treatment, we predicted the activity of transcription factors based on the expression of their target genes using DoRothEA^40^. Subpopulation 3 of the Selumetinib-treated Meta4 sample displayed significant decreases in activity of several transcription factors only in the MEK pathway, including E2f and Ets family members, compared to subpopulation 3 of the DMSO vehicle-treated Meta4 sample (Supplementary Fig. 12B, C). Taken together, these results confirmed the cellular heterogeneity of Meta4 organoids, including subpopulations expressing cancer-related genes.

### DSC populations present in Meta4 have different stemness

From our scRNA-seq data analysis of Meta3 and Meta4 cells, we observed expression of known cancer stem cell marker genes such as CD133 (*Prom1*) and CD166 (*Alcam*)^41^ in both Meta3 and Meta4 (Fig. 2c). To identify whether the cells in Meta3 and Meta4 expressing CD133 and CD166 can be considered pre-cancerous stem cells, we isolated CD133+/CD166+ cells from Meta3 or Meta4 organoids and performed qPCR for metaplastic, stem cell-related and proliferation markers. While the expression of many stem cell-related genes such as *Cd44*, CD133 (*Prom1*) and CD166 (*Alcam*) were significantly enriched in sorted CD133+/CD166+ Meta4 cells, those markers did not show significant changes, except *Aldh1a1* and *Pcna*, in sorted CD133+/CD166+Meta3 cells (Supplementary Fig. 13A, B).

**Fig. 8:**
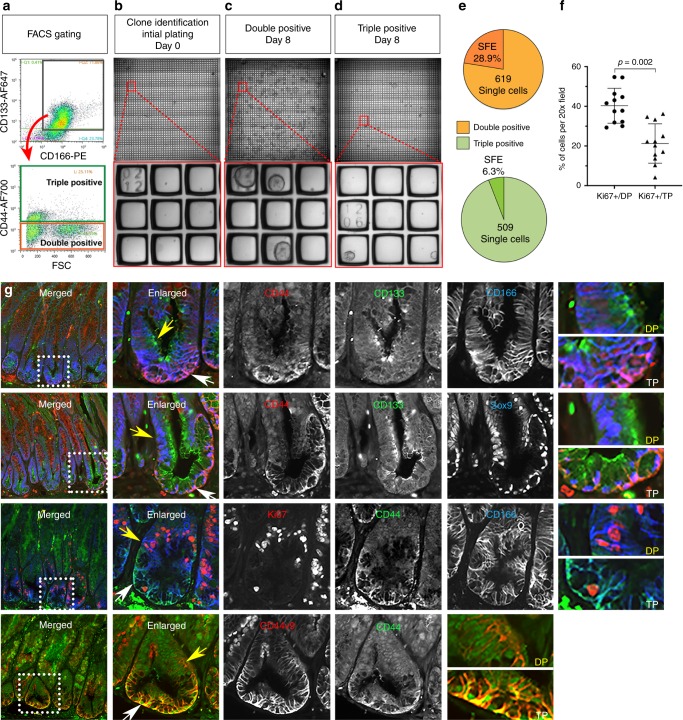
Identification of dysplastic stem cells in Meta4. **a**–**e** Clonal sphere forming efficiency from Meta4 subpopulations. **a** Meta4 organoids were dissociated to single cells, stained for CD44, CD133, CD166, and FACS isolated. Following doublet-discrimination and dead cell exclusion, CD133/CD166 positive cells (gray gate) were applied (red arrow) to a CD44 positive gate to distinguish between double-positive CD44neg/CD133+/CD166+ (orange gate) and triple-positive CD44+/CD133+/CD166+cells (green gate). Double-positive and Triple-positive cells were collected by FACS and applied to CRAs. **b** Immediately after plating, single cells were quantified and their physical address on the CRA was determined. **c**, **d** After 8 days of culture, spheres were quantified and their physical address on the CRA was determined. Upper-panels are low magnification of each CRA containing 2025 wells. Red-boxes indicate the regions in the higher magnification images depicted in the lower panels. **e** Spheres-Forming Efficiency (SFE) of clonal events was determined by quantifying the number of spheres that developed from all single cell events at *t* = 0. There were 619 Double-positive clonal events identified in the CRAs, and 28.9% of those generated spheres (orange chart). There were 509 Triple-positive clonal events identified in the CRAs, and 6.3% of those generated spheres (green chart). The data represent binary outcomes. **f** Quantitation of immunofluorescence staining of Ki67-positive cells in either Double-positive (DP) or Triple-positive (TP) cell zone in Mist1-Kras mouse stomach corpus at 4 months after tamoxifen injection. Data are presented as mean values with standard deviation (*n* = 12). *P*-values were calculated using paired two-tailed *t*-test. **g** Immunostaining for CSC markers, CD44 (basolateral membrane), CD133 (apical membrane) and CD166 (basolateral membrane), metaplasia and gastric cancer markers, Sox9 and CD44v9, and a proliferation marker, Ki67 in Mist1-Kras mice at 4 months after tamoxifen injection. Yellow arrow indicates Double-positive (DP) cells and white arrow indicates Triple-positive (TP) cells. Dotted boxes indicate enlarged area. Scale bars indicate 100 μm. Source data are provided as a Source Data file.

To identify the DSCs and determine the extent of heterogeneity of Meta4 organoids, the Meta4 cells were dissociated and subjected to high-throughput functional analysis by micro-CellRaft Arrays (CRAs)^42,43^. Flow cytometry data demonstrated two populations of DSCs: CD44neg/CD133+/CD166+ (Double-positive) and CD44+/CD133+/CD166+cells (Triple-positive) (Fig. 8a). In contrast, we observed *Cd44* gene expression in both DSC subpopulations from the scRNA-seq data analysis (Fig. 6d), but gene expression data would include all CD44 variant isoforms including CD44v9. The antibody used for sorting recognizes CD44v6, but not CD44v9. Single cells from each population were applied to CRAs and cultured for 8 days to determine clonal survival and sphere forming efficiency (Fig. 8b-d). Figure 8e shows that Double-positive cells, which are negative for CD44, produced clones 4.6-times more efficiently than Triple-positive cells. These results suggest functional differences in stemness between Double-positive and Triple-positive DSCs.

To assess whether the DSCs are also present in vivo, we stained for the markers, which we used for clonal analysis, in the Mist1-Kras mouse stomach corpus at 4 months after tamoxifen injection (Fig. 8g). Based on staining for the expression of CD44, CD133 and CD166 proteins, we identified two DSC subpopulations in vivo: the Triple-positive cells were located at the base of the glands (white arrows) and the Double-positive cells were located right above the Triple-positive cell zone (yellow arrow). Both of the populations expressed Sox9 and CD44v9, validating the same phenotype of DSCs present in Meta4 organoids. Many more Double-positive cells were co-positive for Ki67 compared to Triple-positive cells (Fig. 8f, g). We additionally stained the Mist1-Kras mouse stomach corpus at 3 months after tamoxifen injection using same markers. Although the scRNA-seq data analysis displayed significant expression of the stem cell marker transcripts in Meta3 organoids (Fig. 2c), only CD166 protein was present in the membrane of cells located at the base of IM glands and no expression for CD44 and only weak expression of CD133 were observed in cells of the IM glands (Supplementary Fig. 13C). These results suggest that the Double-positive DSCs represent a more proliferative stem cell population and the Triple-positive DSCs are less proliferative putative stem cell population.

**Fig. 9:**
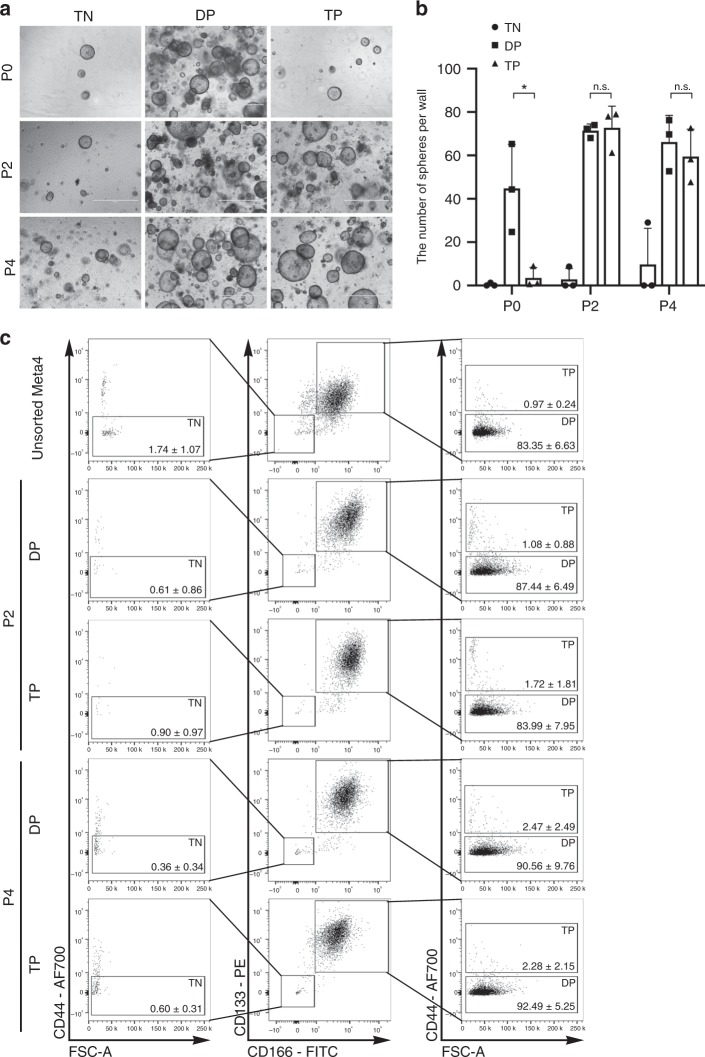
Expansion of spheres derived from single dysplastic stem cells in Meta4. **a** Phase contrast images of spheres derived from isolated Triple-negative (TN), Double-positive (DP) or Triple-positive cells (TP) at passage 0 (P0), 2 (P2) and 4 (P4). Images were captured at about 1 week after plating (P0), at day 5 of P2, at day 5 of P4. TN (CD44neg/CD133neg/CD166neg) cells were isolated as a control population. Scale bars indicate 1000 μm. **b** Quantitation of the number of spheres formed from the Triple-negative (TN), Double-positive (DP) or Triple-positive (TP) cells at P0, P2, and P4. Data are presented as mean values with standard deviation (*n* = 3). *P*-values were calculated using paired two-tailed *t*-test. *P = 0.048 (DP vs. TP at P0); n.s., not significant. **c** Representative FACS profiles of the Triple-negative (TN), Double-positive (DP) and Triple-positive (TP) cells analyzed in unsorted Meta4 organoids and spheres derived from isolated DP or TP cells at P2 and P4. CD44 positive gate was used to distinguish DP and TP cells (right graphs) among CD133+/CD166+cells (top right boxes, middle graphs) or to distinguish TN cells (left graphs) from CD133neg/CD166neg cells (bottom left boxes, middle graphs). The percentage of each TN, DP, and TP cells was not significantly different between unsorted Meta4, DP and TP at P2, and DP and TP at P4. Source data are provided as a Source Data file.

We further examined whether the spheres derived from the isolated DSCs can survive and be propagated. We initially observed fewer numbers of spheres derived from the Triple-positive DSCs and very few spheres from the Triple-negative cells, compared to the number of spheres from Double-positive DSCs, similar to the CRA culture data (Figs. 8e, 9a, b). Interestingly, the spheres from both Double-positive and Triple-positive DSCs were successfully passaged and showed similar sphere expansion rates after passaging (Fig. 9a, b). At passage 2, 87.44 ± 6.49% of cells in the spheres from the Double-positive DSCs sustained the cell fate as Double-positive and 83.99 ± 7.95% of cells in the spheres from the Triple-positive DSCs were Double-positive DSCs. Interestingly, the proportions of the Double-positive DSCs in the spheres either from the Double-positive (90.56 ± 9.76%) or Triple-positive DSCs (92.49 ± 5.25%) were still maintained through passage 4 (Fig. 9c). In addition, Triple-positive DSCs, as well as Triple-negative cells were detected in spheres derived from both Double-positive and Triple-positive DSCs (Fig. 9c). This result implies that the two different stem cell populations can interconvert their cell identity between each other and can undergo terminal differentiation into Triple-negative cells.

## Discussion

In this study we have established gastric metaplastic and dysplastic organoid lines that recapitulate many features of the gastric carcinogenesis cascade observed in mouse models and human patient samples. The organoid lines display distinguishable structures and dynamic behaviors, pathological phenotypes and molecular signatures. Both organoid lines showed active Kras-dependent alterations in cellular behaviors and morphological structures. They have been continually passaged, undergone freezing and thawing following derivation, and maintained distinguishable structures as metaplastic or dysplastic organoids in 3D cultures. The Meta4 organoids demonstrated aggressive characteristics and cellular heterogeneity, including clonogenic growth and engraftment following orthotopic reimplantation into the stomach wall.

Intestinal type-gastric cancer arises within a dynamic metaplastic milieu populated initially by SPEM with progression to IM^5,6^. Since Dr. Correa first identified the association of IM with the development of intestinal type-gastric cancer several decades ago^44^, many studies have sought to understand the cellular mechanisms initiating SPEM development^5,6,8^ and progression to IM in association with chronic inflammation^7,45–47^. However, evaluation of dysplasia, an initial feature of neoplastic changes in gastric carcinogenesis, has been hampered by both a lack of cell lines for gastric metaplasia or dysplasia and an absence of mouse models that develop true neoplasia as seen in humans. The Meta4 organoids showed prominent phenotypes consistent with dysplastic cells. These phenotypic characteristics were driven by active Kras expression in Meta4 cells derived from the Mist1-Kras mouse^5^. However, inhibition of MEK, a downstream mediator of the Kras signaling pathway, led to a remarkable regression of dysplastic structures and behaviors. These insights suggest that active Kras signaling might be one of the dominant signaling cascades driving gastric carcinogenesis from pre-cancerous stages to cancer.

Comparative analysis of transcriptome profiles of Meta3 and Meta4 cells using scRNA-seq also identified distinct molecular signatures in Meta4 cells as dysplastic cells compared to Meta3 cells as metaplastic cells. The Meta4 cells displayed increases in binding or structural molecule activity-related genes including gastric cancer related genes compared to the Meta3 cells. In contrast, Meta4 cells expressed lower levels of IM marker genes such as *Tff3* and *Gpx2* than Meta3 cells, which demonstrate characteristics more consistent with IM. While the Meta4 cells expressed increased cell structure or dysplastic function-related genes, the surviving cells after Selumetinib treatment increased metabolism-related gene expression.

The characteristics of Meta4 cells as dysplasia might be maintained by the two DSC populations. The high-throughput functional analysis using micro-CRAs demonstrated two possible DSC populations, one proliferative and the other relatively less-proliferative. The proliferative DSCs were Double-positive for two cancer stem cell markers, CD133 and CD166, but negative for CD44, which is commonly used to isolate stem cell populations in many tissues^48–50^. In contrast with the expression of CD44v6, which is recognized by the flow sorting antibody, these Double-positive cells do express CD44v9, which is upregulated in metaplasia and early stage gastric cancer and was used to validate Meta4 engraftment (Figs. 3, 8g). Nearly 30% of these Double-positive DSCs were proliferative in clonal survival and spheroid forming efficiency, but both Double-positive and Triple-positive DSC populations displayed flexibility in cell fate conversion and differentiation. We also identified signatures of the two different subpopulations representing proliferative and less-proliferative DSCs in the scRNA-seq data analysis. The proportion of the DSC population by marker expression was increased from 13.2% to 49.3% after the MEK inhibition, however the increased DSC population was not proliferative in surviving Meta4 organoids after MEK inhibition. Also, the surviving dysplastic cells did not sustain continued growth under MEK inhibition, although there were still DSC marker-positive populations. These results suggest that the Double-positive DSCs might be a source for maintaining the dysplastic cell lineages, and stem cell behaviors can be controlled by MEK inhibition. However, all the experiments in this study were performed in vitro using Meta3 and Meta4 organoids. Therefore, the fundamental mechanisms of DSC differentiation into dysplastic cells and their evolution into neoplastic stages, as well as alternative functions or responses of the two DSC populations to extrinsic influences during gastric carcinogenesis in vivo, remain unclear. Also, it is not apparent whether the cells surviving MEK inhibitor treatment are displaying a signature of normal lineage cell differentiation, as we observed in Mist1-Kras mice in our previous study^5^. Therefore, further studies are necessary to elucidate the full spectrum of the functional capacity of the DSCs present in dysplasia as precursors of gastric cancer cells.

Taken together, we have established two organoid lines derived from active Kras-induced mouse stomach corpus. These organoid lines represent an in vitro model system displaying characteristics of intestinalizing metaplasia, as well as dysplasia. Our study provides insights into a role for Kras activation in gastric carcinogenesis and the presence of distinct dysplastic stem cell populations, which might be important for maintaining dysplastic cell lineages and neoplastic transition.

## Methods

### Mice

The generation of Mist1-CreERT2^Tg/+^;LSL-K-ras(G12D)^Tg/+^ (Mist1-Kras), mice has been described previously^5^. Briefly, all the Mist1-Kras mice were maintained on a C57BL/6 background. Five mg of tamoxifen dissolved in corn oil with 10% ethanol was administered to male or female mice at 8 weeks of age by subcutaneous injection, once per day for 3 consecutive days, and mice were sacrificed at 3 months, or 4 months after tamoxifen injection to collect stomach tissues. All mice were housed five per cage in a room with a 12 h light/dark cycle in the Vanderbilt University animal facility. Water and chow were provided ad libitum. The care, maintenance, and treatment of the mice used in this study followed protocols approved by the Institutional Animal Care and Use Committee of Vanderbilt University.

### Gland isolation and organoid generation

Stomach corpus tissues were removed from C57BL/6 wild-type mice or Mist1-Kras mice, cut along the greater curvature of the stomach and washed in ice cold PBS. The antrum tissue area was removed with a razor blade and stomach corpus mucosa was separated from serosa along the muscle layer using cell scrapers. The corpus mucosa was incubated in the pre-warmed digestion buffer (Advanced DMEM/F12 + 5% FBS + 1 mg/mL collagenase type Ia + 1/100 DNAse I) at 37 °C with vigorous shaking at 220 rpm for 30 min and added stopping buffer (Advanced DMEM/F12 + Y-27632 and 1 mM DTT), then strained through 100 μm cell strainer to remove remaining tissue clumps. The dissociated glands were centrifuged at 300×*g* for 5 min to pellet glands, removed supernatant and repeated twice. The supernatant was removed and the pellets were resuspended with ice cold Matrigel (ECM, Sigma). Thirty microliter of gland/Matrigel mixture containing about 100–200 glands was plated in wells of 48 well plates and left the plates at 37 °C incubator for 30 min. Three hundred microliter of Mouse Intesticult (StemCell Technology) medium with a ROCK inhibitor, Y-27632, was added in each well and medium was replaced every 3 days.

### Organoid culture and selumetinib treatment

Selumetinib (AZD6244, Selleckchem) was dissolved in DMSO to obtain 1 mg/mL of stock solutions and stored at −20 °C. Organoids were split and grown in Matrigel without a ROCK inhibitor, Y-27632, for 1–2 days until the organoids grew as spherical structures at around 100 μm in size before Selumetinib treatment. To perform 2D monolayer cultures, the organoids were split and seeded onto 1% Collagen I-coated chamber slides. The organoids were then treated with Intesticult media without Y-27632 containing either DMSO or a final concentration of 1 μM of Selumetinib for 3 days. For passaging organoids after Selumetinib treatment, the organoids were passaged at day 9 with the Intesticult media containing either DMSO or a final concentration of 1 μM of Selumetinib without Y-27632. To perform long-term Selumetinib treatment, the Intesticult media containing either DMSO or a final concentration of 1 μM of Selumetinib were exchanged every 3 days. To assess the effects of Selumetinib treatment on growth, an EVOS FL inverted microscope was used to capture phase contrast images of organoids and to measure the sizes of organoids before and after the Selumetinib treatment. The JuLI^TM^ stage, a Real-Time Cell History Recorder (NanoEntek), was also used to monitor the growth and morphological changes of the organoids in real-time. The phase-contrast images of the organoids were captured every 30 min using the JuLI^TM^ stage at each condition for 3 days and movies were generated. All experiments were run in triplicate and repeated at least two or three times.

### Immunofluorescence staining

Immunostaining for formaldehyde-fixed paraffin-embedded (FFPE) organoids was performed according to a standard protocol. To prepare the FFPE organoids, organoids with Matrigel were fixed in 4% Paraformaldehyde (PFA) at room temperature for 30 min with gentle rocking followed by a wash in PBS at room temperature for 10 min. The fixed organoids with Matrigel were preserved in the HistoGel^TM^ (Thermo Fisher Scientific), then processed according to a standard histological protocol for paraffin embedding. Organoid paraffin sections or Mist1-Kras mouse stomach paraffin sections were de-paraffinized in Histoclear solution (Electron Microscopy Services) and rehydrated in a series of ethanol washes (100%, 95%, 70%). Antigen retrieval was performed using a target retrieval solution, pH 6 (Dako) in a pressure cooker for 15 min. Sections were incubated in Serum-free Protein Block solution (Dako) at room temperature for 1.5 h. Primary antibodies were diluted in Antibody Diluent with background reducing components (Dako), then applied and incubated at 4 °C overnight. After three washes in PBS, secondary antibodies diluted in Antibody Diluent (Dako) were incubated at room temperature for 1 h followed by three washes in PBS. For nuclei counterstaining, sections were incubated in PBS with DAPI (0.2 μg/mL) at room temperature for 5 min, followed by three washes in PBS. All fluorescence images were acquired using a Zeiss Axio Imager M2, equipped with a SPOT Explorer camera using SPOT Basic software. Image overlay and preparation were performed in Adobe Photoshop.

For immunostaining of whole mount organoids, organoids were fixed in 4% PFA at room temperature for 30 min followed by a wash in PBS for 10 min. The organoids were permeabilized using 0.3% Triton X-100 in PBS at room temperature for 30 min followed by blocking with 10% normal donkey serum (NDS) in PBS at room temperature for 1 h followed by three washes in PBS for 10 min each. Primary antibodies were diluted in PBS with 1% NDS and incubated at 4 °C for overnight followed three washes in PBS containing 0.1% Tween 20 (PBS-T) for 20 min each. Secondary antibodies were diluted in PBS with 1% NDS and incubated at room temperature for 2 h followed by three washes in PBS-T for 20 min each. For nuclei counterstaining, DAPI (0.2 mg/mL) in PBS was added and incubated at room temperature for 5 min followed by three washes in PBS-T. ProLong Antifade Reagent was added to wells and organoids imaged using either a Zeiss LSM 710 or a Nikon A1R confocal microscope. Primary antbodies used were: CD444v9 anti-rat (Cosmo Bio CAC-LKG-M002, 1:25,000); Cdx1 anti-rabbit (Thermo Scientific PA5-23056, 1:500); Sox9 anti-Rabbit (Millipore Ab5535, 1:1500); CD44 anti-mouse (Invitrogen MA5-15462, 8E2F3, 1:1000); CD133 anti-rat (eBioscience 14-1331-80, 13A4, 1:100); CD166 anti-rabbit (Abcam ab109215, 1:250); Ki67 anti-rat (Biolegend 652402, 16A8, 1:200); Villin-1 anti-rabbit (Cell Signaling Technology 2369, 1:300); Vimentin anti-mouse (Sigma-Aldrich V6630, V9, 1:300); Pan-CK anti-rabbit (Dako Z0622, 1:4000); Phospho-Ezrin anti-rabbit (Cell Signaling Technology 3726, 48G2, 1:300); Cortactin anti-mouse (Millipore 05-180, clone 4F11, 1:1000); Phospho-ERK1/2 anti-rabbit (Cell Signaling Technology 4370S, 1:2000); RFP anti-rabbit (Rockland 600-401-379, 1:500); FITC-conjugated UEAI-lectin (Sigma L9006, 1:2000); Alexa Fluor™ 647 Phalloidin (Invitrogen A22287, 1:100). Species-specific secondary antibodies (1:500) were purchased from ThermoFisher and details are in the Supplementary Table 1.

### Orthotopic implantation

Meta4 organoids were washed in ice cold PBS to remove Matrigel and approximately 100 organoids were mixed with 50 μL of Cultrex BME (Cultrex® Basement Membrane Matrix, Type 3). Before surgery, the mice were treated with subcutaneous injection of a dose of buprenorphine hydrochloride (0.05 mg/kg; Temgesic, BD Pharmaceutical System). Both male and female C57BL/6 mice were used for the experiment. Eight-week old mice were sedated with 2% isoflurane inhalation anesthesia. The stomach was exposed through a small abdominal incision and the BME mixture containing organoids was injected into the stomach muscularis layer using a 26 G × 3/8 inch syringe needle (BD Pharmaceutical system), and then the stomach was replaced into the abdominal cavity. After surgery, the mice were treated with subcutaneous injection of 3 doses of buprenorphine hydrochloride (0.05 mg/kg; BD Pharmaceutical System) and of ketoprofen (5 mg/kg; BD Pharmaceutical System). All survival surgery procedures followed protocols approved by the Institutional Animal Care and Use Committee of Vanderbilt University.

### FACS

Meta4 organoids were first cultured and passaged in 30 μL of Matrigel with 300 μL Mouse Intesticult media in a 48-well tissue culture plate. Fifteen to twenty wells of the Meta4 organoids were dissociated to single cells for FACS by removing media, and washing each Matrigel plug with 1 mL of PBS for 1 min each, then 500 μL of TrypLE (Thermo Fisher, USA) was added to each well. The Meta4 in TrypLE were consolidated by transferring to a 15 mL conical tube. An additional 500 μL of TrypLE was used to rinse all wells to collect all remnants of organoids. The Meta4 in TrypLE were incubated between 11 and 12 min in a 37 °C water bath, and triturated every 2–3 min to assist in breaking up cells. This time was empirically determined to produce the most single cells without compromising cell viability. The TrypLE was quenched using 300 μL of FetalPlex serum (Gemini Bioproducts, USA) and Advanced DMEM (Thermo Fisher, USA) was added to a 15 mL total volume to washing cells. The tube was held on ice for 10 min, and the cells were pelleted at 500×*g* for 5 min at 4 °C. The media was removed, and cells were resuspended in 1.5 mL of Mouse Intesticult with 1% FetalPlex, and kept on ice until antibody staining for FACS. Single color controls were set up by aliquoting 25 μL of the cell suspension into a 1.5 mL tube, and adding 1 μL of antibody or live/dead stains. For live/dead cell detection SytoxBlue and AnnexinV-PacBlue were used. For cell sorting, all antibodies were added to the remaining cell suspension at 1:100 dilution and incubated on ice for 45 min. The live/dead stains were not added until immediately prior to FACS. Cells were washed by adding Advanced DMEM with 10 µM Y27632 (wash media) to a 1.5 mL volume and pelleting at 2000×*g* for 3 min. The staining media was aspirated, and the cells were washed once more as described with 1.5 mL of wash media. The cells to be sorted were resuspended in 500 μL of Mouse Intesticult with 10 µM Y27632, and live/dead stains were added at 1:1000 for SytoxBlue, and 1:100 for AnnexinV-PacBlue. Single color controls were used to determine positive and negative staining and to establish gating parameters for sorting. Double positive (CD44−, CD133+, CD166+), Triple positive (CD44+, CD133+, CD166+) or Triple negative (CD44−, CD133−, CD166−) cells were sorted into separate tubes using a Sony SH800 FACS instrument or a FACSAria III (BD Biosciences). Five thousand cells each were plated in wells of 48-well plates and the plates were maintained at 37 °C in an incubator for 30 min. Three hundred microliter of Mouse Intesticult (StemCell Technology) medium was then added in each well and was replaced every 3 days. Once the cells grew as spherical structures, the spheres were passaged. For FACS analysis, the spheres were dissociated 5 to 7 days after passaging, before the spheres began budding formation, and stained with antibodies following the same protocol used for the cell sorting. Gating strategy is indicated in Supplementary Fig. 14. The cells were analyzed using a LSR II flow cytometer (BD Biosciences). All experiments were repeated three times. Antibodies and stains used for FACS were Alexa Fluor® 700-CD44 anti-mouse (Biolegend 338813, 5 µL/10^6^ cells in 100 µL); Alexa Fluor® 647-CD133 anti-rat (Biolegend 141215, 0.5 mg/mL); PE-CD133 anti-rat (Biolegend 141204, Clone 315-2C11, 0.2 mg/mL); PE-CD166 anti-goat (R&D systems FAB1172P, 10 µL/10^6^ cells); Fluorescein-CD166 anti-goat (R&D systems, FAB1172F, 10 µL/10^6^ cells).

### Soft agar assay

0.8% melted agarose gel was added onto wells of 24 well plates as a base layer and solidified at 4 °C for about 5 min. About 50 organoids were mixed with 250 μL of 0.48% melted agarose and overlaid onto the solidified base layer, followed by incubation at 4 °C for about 5 min. Mouse Intesticult media containing with either DMSO or Selumetinib was added to wells and incubated at 37 °C for two weeks. Media was exchanged every three days. The JuLI^TM^ Stage was used for live imaging of colony formation for first 3 days and the experiment was continued for a total of 2 weeks. GelCount^TM^ was used to count colony numbers before and after the assay. The organoids were then fixed in 4% PFA at room temperature for 30 min, followed by a wash in PBS for 10 min. The fixed organoids preserved in agarose were then processed according to a standard histological protocol for paraffin embedding.

### inDrop single cell RNA sequencing

Organoids from 5–8 wells in 48-well plates were pooled together by removing media and washing each Matrigel plug with 1 mL of cold PBS for 1 min each, followed by dissociation into single-cell suspensions using TrypLE (gibco). Single-cell encapsulation and sequencing was performed as previously reported^29,32^. Briefly, cell viability was determined by counting Trypan Blue positive cells. The single-cell suspension was further enriched with a MACS dead cell removal kit (Miltenyi) prior to encapsulation and the density of cells was calculated by counting. Single cells were encapsulated and barcoded using the inDrop platform (1CellBio) with an in vitro transcription library preparation protocol^4,5^. After library preparation, the samples were sequenced using Nextseq 500 (Illumina) using a 150 bp paired-end sequencing kit in a customized sequencing run (50 cycles read 2, 6 for the index read, rest for read 1). Samples were multiplexed in a single sequencing run.

After sequencing, reads were filtered, sorted by their barcode of origin, and aligned to the reference transcriptome using the inDrop pipeline^5,30^. Mapped reads were quantified into UMI-filtered counts per gene and barcodes that correspond to high-quality cells were retrieved based on the previously established inflection point method, resulting in the pre-processed data table^31^. This resulted in 500 cells from the Meta3 sample, 500 cells from the Meta4 sample, 2471 cells from the DMSO-treated Meta4 sample, and 905 cells from the Selumetinib-treated Meta4 sample. The accession numbers for the data reported in this paper are NCBI Gene Expression Omnibus (GEO): GSE121940.

Analysis of pre-processed scRNA-seq data was carried out in RStudio version 3.5.2 and Seurat version 3.0.0 (refs. ^33,34^). Functions use default arguments unless specified. Because the samples were encapsulated together with the inDrop platform and sequenced together in the same lane, we assumed no batch effects, as we have shown in a previous experimental design, and therefore, no batch corrections were performed ^2^.

The following steps were performed separately for each pre-processed sample or, when appropriate, combined samples as previously described^7,33^. A Seurat object was created for the individual and combined samples using Seurat’s CreateSeuratObject function. The number of genes, number of UMIs, and percent mitochondrial expression for all cells in each sample was visualized (Supplementary Fig. 3A and 8A, B). The NormalizeData, FindVariableFeatures (vst method with 2000 features), and ScaleData Seurat functions were used to normalize, scale and center, and to find highly variable genes (HVGs) (Supplementary Fig. 8C). HVGs were not used for Meta3 and Meta4 samples. Principle component analysis (PCA) was performed using Seurat’s RunPCA function using only HVGs as features for dimension reduction when appropriate (Supplementary Figs. 3B-C, 8D-E, 9A-B, E-F). The jackstraw method (Seurat’s JackStraw function with 30 PCs) or the ElbowPlot function was used to determine the PCs to use for UMAP (Seurat’s RunUMAP function) or t-SNE visualization (Seurat’s RunTSNE function)^37,51^ (Figs. 2a, 6a and Supplementary Figs. 4A,C,E, 8F, 9C-D, G-H). Seurat’s FindNeighbors and FindClusters functions were used for clustering (Figs. 2a, 6a and Supplementary Figs. 4A,C,E, 8G) and subpopulation-matching was performed for DMSO vehicle-treated and Selumetinib-treated Meta4 samples, as detailed below. Differential expression analysis was performed using Seurat’s FindMarkers and FindAllMarkers functions. To determine the appropriate number of clusters in each sample, the clustering resolution parameter was adjusted and differential expression analysis was performed on all clusters—the appropriate number of clusters is the minimum number of clusters with differentially expressed genes in all subpopulations (Supplementary Figs. 4A-F, 9D,H). Cell cycle genes were selected based on the lists published in Fig. 2 and Table 2 by Whitfield et al.^39^. To determine activity for each phase of the cell cycle, library size-normalized count data was then normalized gene-wise for cell cycle genes. Next, the average expression of these normalized values for cell cycle genes within each phase (G1-S, S, G2, G2-M, and M-G1) was calculated and a meta-gene for each phase was created. Thresholds for differentially expressed genes are reported in figure legends.

Seurat’s clustering methods did not identify matched subpopulations in DMSO-treated and Selumetinib-treated samples and clustering inappropriately identified more subpopulations in the DMSO-treated sample than in the Selumetinib-treated sample (Supplementary Fig. 8G). Therefore, we developed an algorithm that matches subpopulations across a perturbation, such as drug treatment, in order to identify similar subpopulations based off gene expression signatures. We used clustering results on individual samples (DMSO-treated and Selumetinib-treated) from the Seurat pipeline (Supplementary Fig. 9D,H) and matched these subpopulations between samples based off gene signatures. The following steps were performed for each dataset (DMSO-treated or Selumetinib-treated Meta4 samples) separately until specified. Counts form pre-processed data tables were normalized by library size for single-cells, multiplied by a factor of 1000, and transformed by computing the hyperbolic sine. Subpopulations identities were assigned arbitrary color identities to visualize them in t-SNE space (Supplementary Fig. 10A). Next, a 2-sample students t-test assuming unequal variance and unpaired samples was used to find significant differences in genes across subpopulations within a sample. For each gene, all cells within a subpopulation were compared to the remaining cells in the sample using the transformed data. Next, the fold change for each gene was found in a similar fashion using transformed data, where the fold change is the arithmetic difference between the mean of the cells within the subpopulation and the mean of the remaining cells, for each gene. In order to identify which fold changes were significant, the p-values calculated previously were used with a significance threshold of 0.001. These steps allow identification of significantly different fold changes for each subpopulation and each gene. The fold changes were discretized to values of 1, −1, and 0 to indicate significantly upregulated genes, significantly downregulated genes, and non-significant genes, respectively. Together, these discrete values make up the gene signature for each subpopulation within DMSO-treated or Selumetinib-treated Meta4 samples. To compare gene signatures in all subpopulations across the samples, we determined if the discretized values are identical between subpopulations, which is considered a match. Matches are determined for all genes from all combinations of samples, and the total number of matches are summed for each of these combinations. In order to identify if these matches are significant, a permutation approach was used. The gene signatures for the DMSO-treated sample were randomized 100 times using the randomizeMatrix function from the picante R package. Next, these 100 permutations of DMSO-treated subpopulation gene signatures were compared to all Selumetinib-treated subpopulation gene signatures, as described before. This yielded a distribution of 100 values for the number of matches that are expected to occur by chance. This allowed us to determine if the number of actual matches between subpopulations in samples is significantly greater than the number of matches that occur by chance (threshold of 4 standard deviations), indicating the subpopulations from the two samples are similar cell types or states (Supplementary Fig. 10A). The gene signatures for these matched subpopulations are those genes that matched across treatment and are visualized in a heat map using the pheatmap R package (Supplementary Fig. 10B). This method determines, in a quantitative manner, if cell types (i.e., subpopulations) are conserved across a treatment or condition, and additionally, identifies a gene signature for those subpopulations that are matched. To confirm that the subpopulation-matching method is robust, we performed differential expression analysis for each matched-subpopulation, as described above, and visualized the results (Supplementary Fig. 10C). These matched subpopulations were used to analyze the DMSO-treated and Selumetinib-treated Meta4 samples (Fig. 6a).

Software and algorithms used for scRNA-seq data analysis are reported in Supplementary Table 2.

Transcription factor activity was estimated for single cells in DMSO-treated and Selumetinib-treated samples using the DoRothEA package (v2) developed by Garcia-Alonso et al.^40^. Only transcription factor-target interactions at the highest confidence score (A) were used in our analysis. Statistical comparisons were made between matched subpopulations in DMSO-treated vs. Selumetinib-treated samples. Statistically significant results are reported as those with a signal to noise ratio of >0.4 and an adjusted *p*-value (Wilcoxon test with Benjami-Hochberg multiple testing correction for all transcription factor activities) >0.01.

### PCR

Total RNAs were extracted from Meta4 organoids treated with ether DMSO vehicle or 1 μM Selumetinib for 3 days using Trizol (Invitrogen), and cDNAs were synthesized using iScript reverse Transcription Supermix (Bio-Rad). Triplicate cDNA samples were used to perform PCR using DreamTaq Green PCR Master Mix (Thermo Scientific) or GoTaq Green Master Mix (Promega). For DreamTaq, the PCR condition was as follow: 94 °C for 2 min, 35 cycles of 94 °C for 30 s, 55 °C for 30 s and 72 °C for 30 s, and a final extension at 72 °C for 2 min. For GoTaq, the PCR condition was as follow: 94 °C for 2 min, 35 cycles of 94 °C for 30 s, 55 °C for 30 s and 72 °C for 1 min, and a final extension at 72 °C for 10 min. The primer sequences are indicated in Supplementary Table 1.

### Quantitative real-time PCR

Meta3 or Meta4 organoids treated with either DMSO vehicle or 1 μM Selumetinib for 3 days and sorted by FACS were used to extract total RNAs and cDNAs were synthesized using iScript reverse Transcription Supermix (Bio-Rad). Triplicate cDNA samples were used to perform quantitative real-time PCR (qPCR) using SsoAdvanced^TM^ Universal SYBR Green supermix and a CFX96 Real-Time PCR Detection System (Bio-Rad). The primer sequences used are shown in Supplementary Table 1 and data was analyzed using CFX Maestro software (Bio-Rad).

### Western blot

Proteins were extracted from Meta4 organoids treated with either DMSO vehicle or 1 μM Selumetinib for 1 day using M-PER lysis buffer (Thermo Fisher Scientific) with protease and phosphatase inhibitor cocktails. The protein concentration was measured by the Direct Detect IR spectrometer (Millipore). Eight μg of total protein were loaded onto a Mini-Protean TGX Precast Gel (Bio-Rad) and transferred to a nitrocellulose membrane (Bio-Rad). The membranes were blocked with the Odyssey blocking solution (LI-COR Biosciences) for 1 h at room temperature and incubated overnight at 4 °C with primary antibodies diluted in the Odyssey blocking solution (LI-COR Biosciences) supplemented with 0.2% Tween-20. Primary antibodies used for western blot were ERK1/2 anti-rabbit (Cell Signaling Technology 4695, 1:1000); Phospho-p44/42 MAPK (Erk1/2) anti-rabbit (Cell Signaling Technology 4370S, D13.14.4E, 1:2000); β-Actin anti-mouse (Sigma A5316, AC-74, 1:2500). After primary antibody incubation, the membranes were washed three times in TBS-T and incubated with IRDye 800CW donkey anti-rabbit (LI-COR Biosciences 926-32213, 1:15,000) or IRDye 680LT donkey anti-mouse secondary antibody (LI-COR Biosciences 926-68022, 1:15,000) for 1 h at room temperature. The membranes were washed three times with TBS-T and imaged with an Odyssey imaging system (LI-COR Biociences).

### Transmission electron microscopy

Organoid samples were prepared and processed by the Vanderbilt Cell Imaging Shared Resource, Vanderbilt University. Briefly, Meta4 organoids treated with either DMSO vehicle or Selumetinib (1 μM) were washed in 0.1 mol/L cacodylate buffer and fixed in buffer (2% paraformaldehyde, 2.5% glutaraldehyde, and 0.1 mol/L cacodylate buffer) at room temperature (RT) for 1 h, then at 4 °C for 4 days or overnight. Organoids were washed with 0.1 mol/L cacodylate buffer at room temperature for 10 min, treated with 1% osmium tetroxide in 0.1 mol/L cacodylate buffer for 1 h at RT, and washed with 0.1 mol/L cacodylate buffer for 10 min at RT, then with distilled water for 10 min at RT. Samples were subjected to 2% uranyl acetate *en* bloc for 30 min for additional contrast. The samples were then washed in water before proceeding to ethanol dehydrations. Organoids were dehydrated through an ethanol series (from 30 to 100%), then incubated for 5 min in 100% ethanol and propylene oxide (PO), followed by two exchanges of pure PO. The organoids were infiltrated with Epon 812 resin (Electron Microscopy Sciences, Hatfield, PA) and PO (1:3) for 30 min at RT, and then infiltrated with Epon 812 resin and PO (1:1) for overnight at RT. Next day, the samples went through a resin:PO (3:1) exchange for 3 to 4 h, and then were incubated with pure epoxy resin overnight. The next day, the organoids were incubated in two more changes of pure epoxy resin, then allowed to polymerize at 60 °C for 48 h. Ultrathin sections (70 to 80 nm thick) were cut and collected on 300-mesh copper grids. The sections were stained with 2% uranyl acetate and then with Reynold’s lead citrate. The organoids were observed using a Philips/FEI T-12 Tecnai T12 electron microscope (Vanderbilt Cell Imaging Shared Resource, Vanderbilt University, Nashville, TN).

### CellRaft array culture and analyses

Five thousand double-positive or triple positive cells were applied in 1 mL of Advanced DMEM with 10 µM Y27632 into a CellRaft Array (CRA) (Cell Microsystems, USA) containing 2025 microwells (CRA quad-well) each with 200 µm^2^ dimensions. This number of cells produced between 500–600 single cells per well. The CRAs were centrifuged at 10×*g* at 4 °C for 5 min to ensure that each cell was at the bottom of the well. The media was carefully removed and replaced with 250 µL of Matrigel. CRAs were re-centrifuged at 4 °C for 5 min to ensure cells remained at the bottom of the wells. The CRAs were carefully placed at 37 °C for 20 min to polymerize the Matrigel, and then 500 µL of Mouse Intesticult was added to each well of the quad-well array. The arrays were tile imaged at 40×-original magnification immediately after initial plating, and then again at Day 8 after plating when fully developed gastroids formed. CRAs were incubated at 37 °C under 5% CO_2_ and a humidified atmosphere. Intesticult media without Y27632 was replaced every two days. Meta4 sphere forming efficiency (SFE) was determined by quantifying the number of Meta4 at Day 8 that developed from single cells.

### Statistics and reproducibility

For quantitation of budding organoids in Meta3 and Meta4, a total of 50 to 100 organoids were considered from three representative images taken from three wells of each organoid line at ×4 magnification. The number of organoids with budding structures were manually counted. For measurement of organoid size after Selumetinib treatment, a total of 75 organoids were used from two representative images taken from three wells of each condition at ×4 magnification. The experiments were repeated twice. Organoids showing their entire area in the images were only considered for measurement and diameters were manually measured for each organoid. For counting colony numbers after the soft agar assay, colonies of at least 100 μm diameter from three wells of each treatment condition were counted using Gelcounter software and the experiment was repeated three times. The average values from each condition were compared by One-way ANOVA, paired *t*-test or Wilcoxon signed-rank analyses for multiple comparison using Graphpad Prism. *P < 0.05, **P < 0.005, ***P < 0.0005, ****P < 0.0001. scRNA-seq statistical thresholds and methods are specified in the respective methods section and figure legends. All experiments were repeated at least two times.

### Reporting summary

Further information on research design is available in the Nature Research Reporting Summary linked to this article.

## Supplementary information

Supplementary Information

Supplementary Movie 1

Supplementary Movie 2

Supplementary Data 1

Reporting Summary

Description of Additional Supplementary Files

## Data Availability

The authors declare that all data supporting the findings of this study are available within the article and its supplementary information files or from the corresponding author upon reasonable request. Two scRNA-seq data sets that support the findings of this study have been deposited in the NCBI Gene Expression Omnibus (GEO) database under accession code: GSE121940 . The source data underlying Figs. 1E, 3, 4B, D, 5A-C, 8F, G, 9B, C and Supplementary Figs. 1E, 2A, B, 5B, 6A, B, E, F, 7B, D, 13A-C are provided as a Source Data file.

## Source data

Source Data
