# Supplementary material for: Heterogeneity and dynamics of active Kras-induced dysplastic lineages from mouse corpus stomach
Source: Nat Commun. 2019 Dec 5;10:5549. doi: 10.1038/s41467-019-13479-6 (PMC6895174; doi:10.1038/s41467-019-13479-6)
Supplement: Supplementary file 1 — Supplementary Information [file 41467_2019_13479_MOESM1_ESM.pdf]

Supplementary Figures, Tables and References

Heterogeneity and dynamics of active Kras-induced dysplastic lineages from mouse corpus stomach

Min and Vega et al.

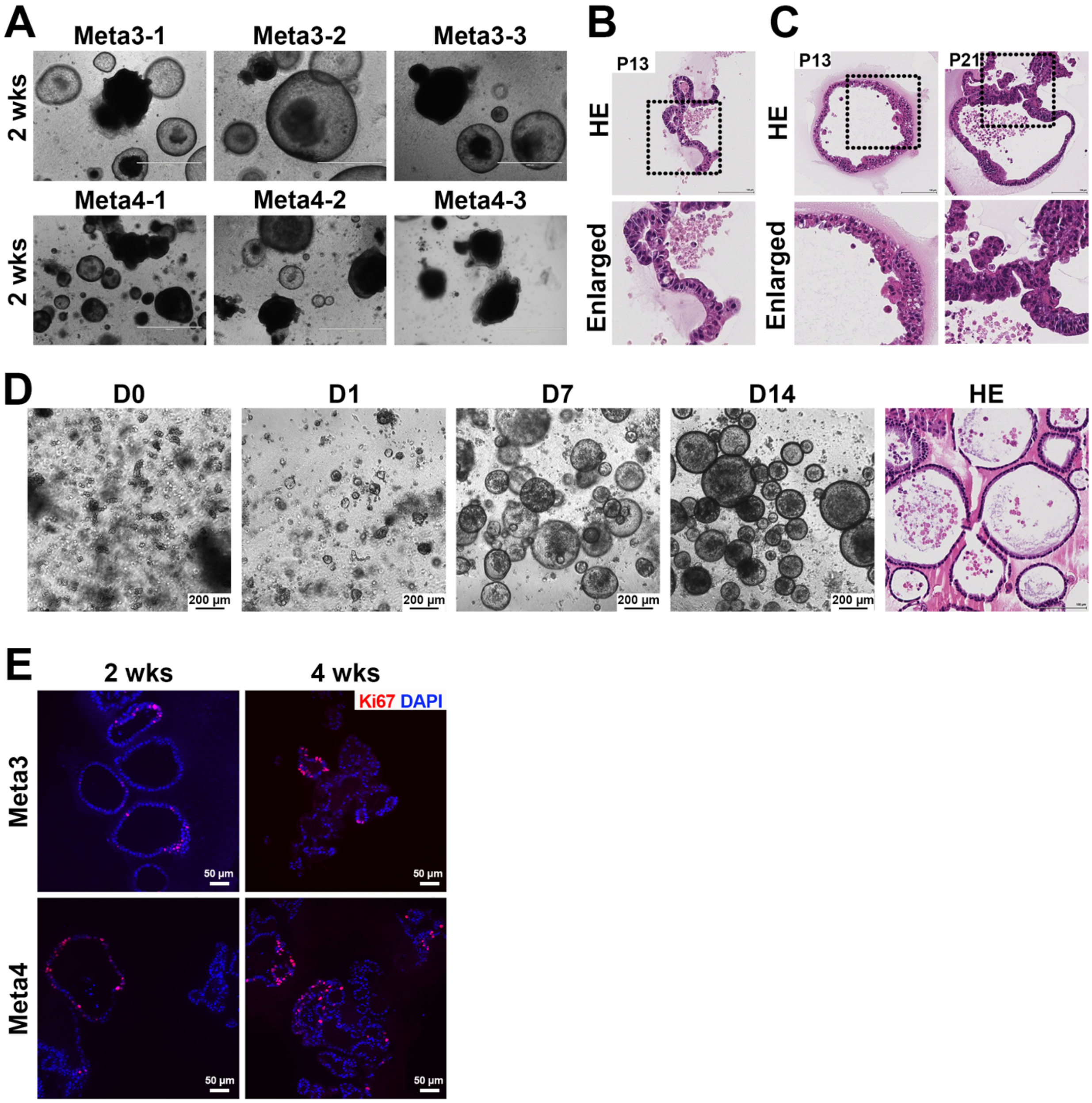

Supplementary Figure 1. Examination of Meta3 and Meta4 organoids and normal gastric gland culture isolated from wild-type mouse stomach corpus. A) Phase contrast images of three Meta3 lines and three Meta4 lines captured 2 weeks after plating in Matrigel. Scale bars indicate 1000 μm. B-C) Paraffin embedded sections from Meta3 organoids at passage 13 (P13) and Meta4 organoids at passage 13 and 21 (P13 and P21). Dotted boxes indicate enlarged area. Scale bars indicate 100 μm. D) Phase contrast images of normal gastroids captured 0, 1, 7, 14 days after plating in Matrigel and paraffin embedded sections from normal gastroids at 2 weeks in 3D culture examined by H&E staining. No significant budding structures were observed in normal gastroids. Scale bars indicate 200 μm in phase contrast images and 100 μm in the H&E image. E) Immunostaining for Ki67 (red) in Meta3 and Meta4 at 2 and 4 weeks of cultures in 3D. Nuclei were counterstained with DAPI (blue). Scale bars indicate 50 μm. Source data are provided as a Source Data file.

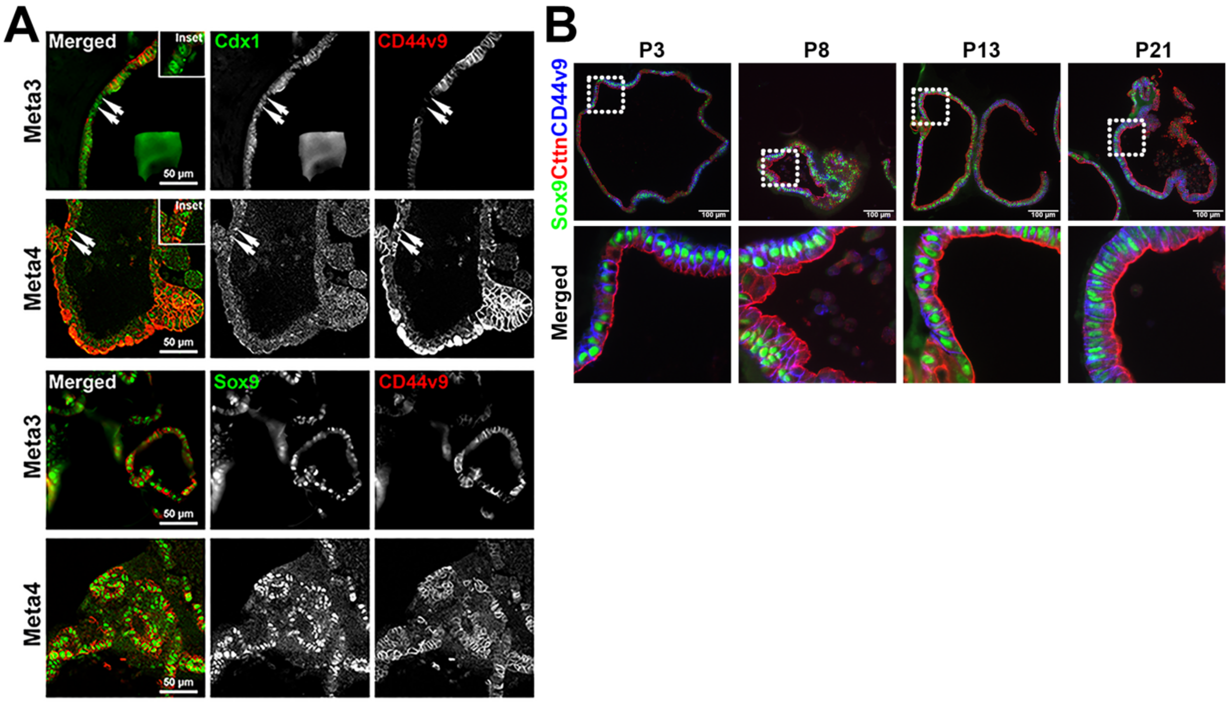

Supplementary Figure 2. Immunostaining for IM or SPEM and early stage gastric cancer markers. A) Paraffin embedded Meta3 and Meta4 organoids were immunostained for an IM marker, Cdx1 (green) or SPEM and early stage gastric cancer markers, Sox9 and CD44v9. White arrows indicate Cdx1 positive cells. Scale bars indicate 50 μm. B) Co-immunostaining for markers which are present in Meta4, Sox9, Cortactin (Cttn) and CD44v9, in paraffin sections of Meta4 at different passages (P3, P8, P13 and P21). Scale bars indicate 100 μm. Source data are provided as a Source Data file.

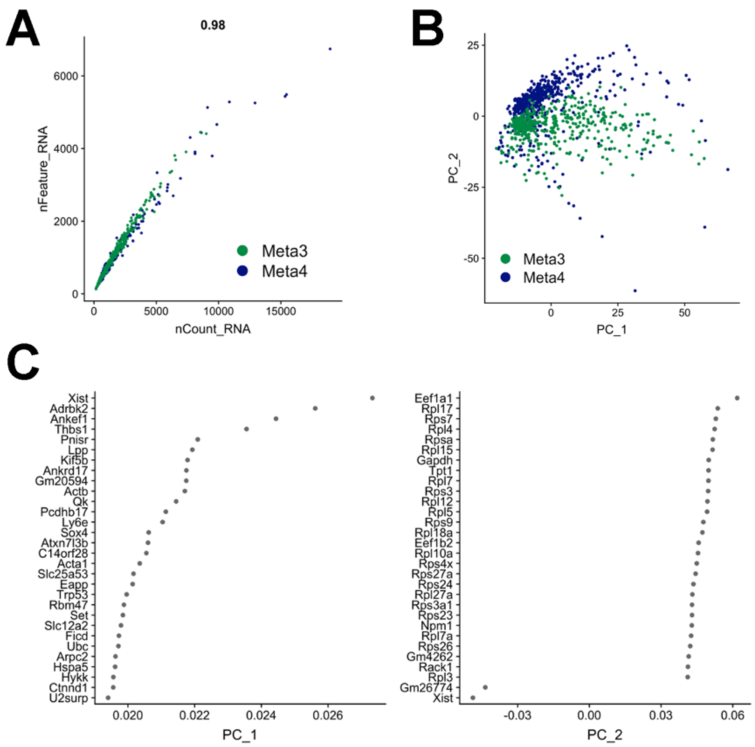

Supplementary Figure 3. Seurat pipeline used to analyze Meta3 and Meta4 samples. A) Number of genes/features (y-axis) versus number of counts/UMIs (x-axis) for Meta3 and Meta4 samples. B) Principle-component (PC) 2 versus 1 for Meta3 and Meta4 samples analyzed together. C) Visualization of the top genes associated with the first 2 PCs for Meta3 and Meta4 samples analyzed together.

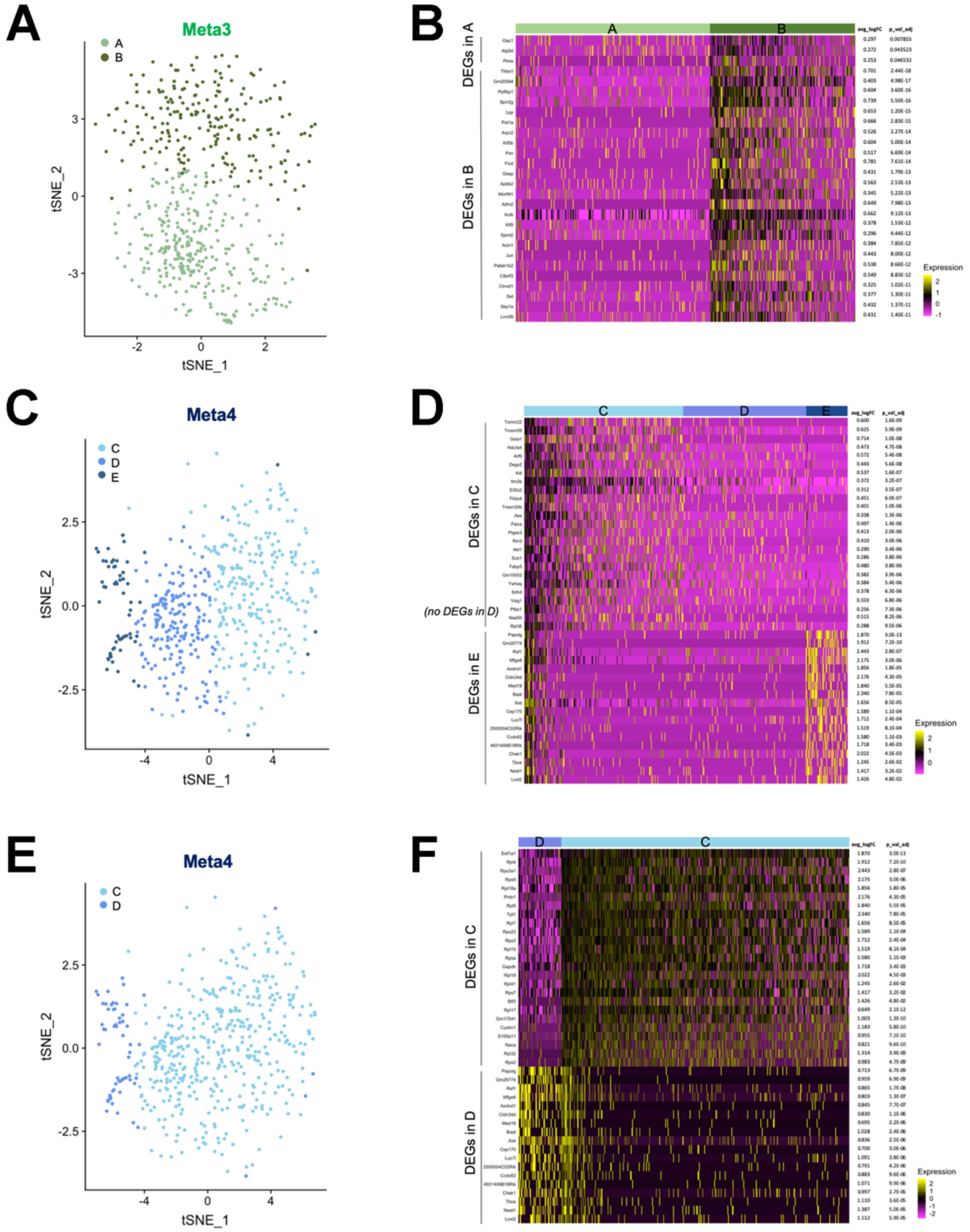

Supplementary Figure 4. Clustering and differential expression analysis of Meta3 and Meta4 samples. A, C, E) t-SNE dimension reduction results with Seurat clustering on Meta3 sample alone with clustering resolution = 0.2 (A), Meta4 sample alone with clustering resolution = 0.3 (C), and Meta4 sample alone with clustering resolution = 0.2 (E). B, D, F) Heatmap of approximately 25 of the most differentially expressed genes (DEGs) for each subpopulation based on clustering results in A, C, or E, respectively. DEGs were defined as those expressed in at least 25% of the cells in the cluster with at least +0.25 log fold-change over all other cells. Adjusted p-values were calculated using a two-tailed Wilcoxon Rank Sum test with Bonferroni correction and were <0.05. Rows correspond to individual genes and columns are individual cells, arranged by subpopulation. Yellow corresponds to high expression, black corresponds to neither high nor low expression, and purple corresponds to low expression.

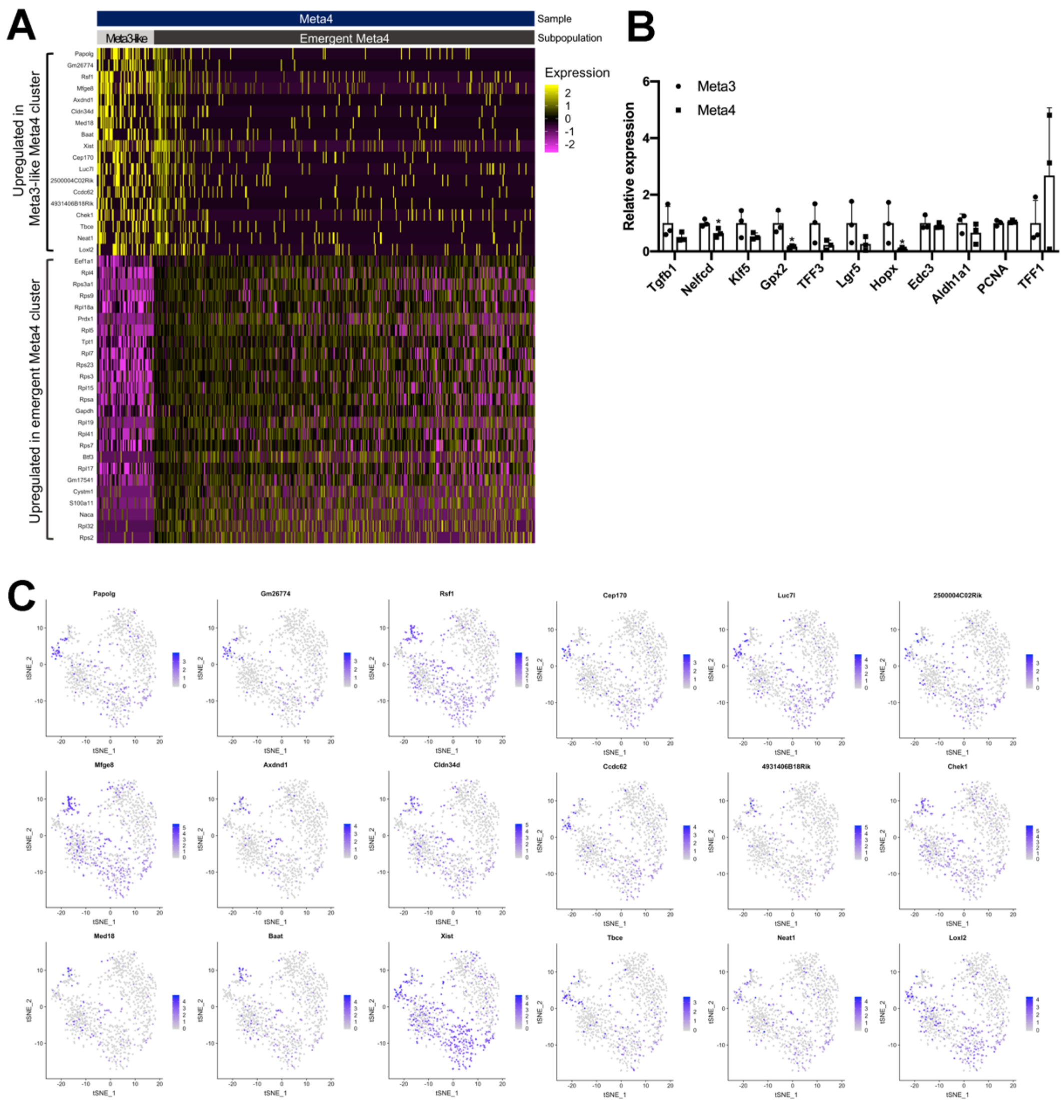

Supplementary Figure 5. Differential expression analysis in Meta4 subpopulations suggests Meta3-like Meta4 and emergent Meta4 subpopulations. A) Heatmap of approximately 25 of the most upregulated genes in each subpopulation (Meta3-like Meta4 and emergent Meta4) of the Meta4 sample. Upregulated genes were defined as those expressed in at least 25% of the cells in the cluster with at least 0.1 log fold-change over all other cells. P-values were calculated using a two-tailed Wilcoxon Rank Sum test with Bonferroni correction and were <0.05. Rows correspond to individual genes and columns are individual cells, arranged by subpopulation. Yellow corresponds to high expression, black corresponds to neither high nor low expression, and purple corresponds to low expression. B) Quantitative PCR showing relative expression levels of several differentially expressed genes between Meta3 and Meta4 which identified in scRNAseq analysis. Expression levels were normalized to TBP gene. Data are presented as mean values with standard deviation (n=3). P-values were calculated using unpaired one-tailed t-test. * P<0.05 (0.11; *Tgfb1*, 0.02; *Nelfcd*, 0.09; *Klf5*, 0.01; *Gpx2*, 0.07; *TFF3*, 0.09; *Lgr5*, 0.047; *Hopx*, 0.29; *Edc3*, 0.15; *Aldh1a1*, 0.24; *PCNA*, 0.16; *TFF1*). Source data are provided as a Source Data file. C) t-SNE plots of Meta3 and Meta4 samples. Relative expression of differentially expressed genes from the Meta3-like Meta4 subpopulation compared to the emergent Meta4 subpopulation are overlayed, as indicated by color.

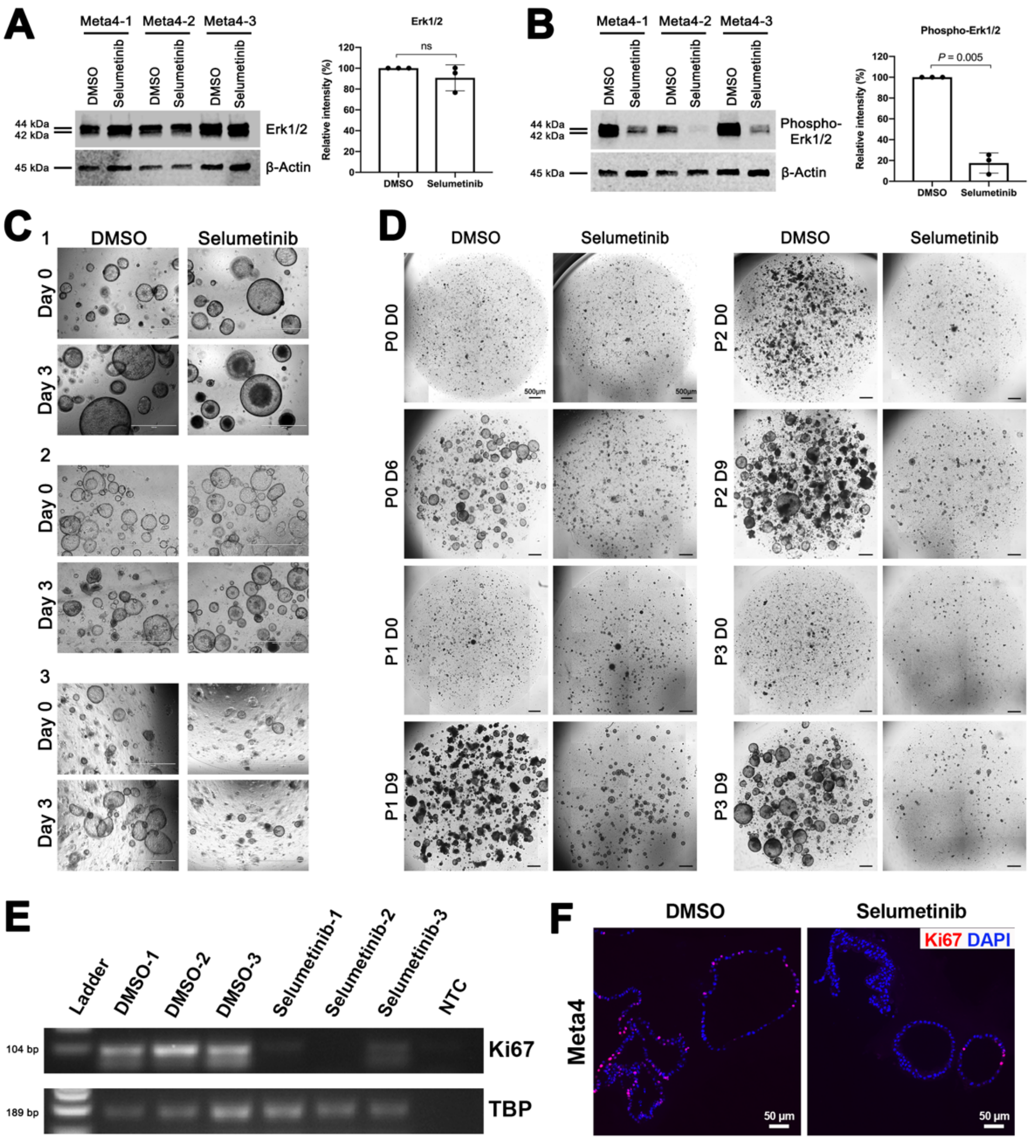

Supplementary Figure 6. Examination of the effects of MEK inhibition in Meta4 organoids. A-B) Western blot for detection of total Erk1/2 (A) and phospho-Erk1/2 (B) protein in three different Meta4 organoid lines treated with either DMSO vehicle or Selumetinib (1μM) for 1 day. β-Actin was used as a loading control. Data are presented as mean values with standard deviation. P-values were calculated using paired two-tailed t-test. C) Three Meta4 organoid lines were treated with either DMSO containing control media or Selumetinib (1μM) containing media for 3 days. Phase contrast images were captured before and 3 days after the DMSO vehicle or Selumetinib treatment. Scale bars indicate 1000 μm. D) Meta4 organoids were treated with either DMSO vehicle or Selumetinib (1μM) for 6 days, then passaged with either DMSO vehicle or Selumetinib (1μM) containing media three times. Phase contrast images were captured before and 6 or 9 days after the DMSO vehicle or Selumetinib (1μM) treatment. Scale bars indicate 500 μm. E) RT-PCR to measure Ki67 gene expression levels in three DMSO vehicle-treated and three Selumetinib-treated Meta4 organoids. TBP was used as a control. NTC; non-template control. F) Immunostaining for Ki67 (red) in paraffin sections of Meta4 treated with either DMSO vehicle or Selumetinib for 3 days. Nuclei were counterstained with DAPI (blue). Scale bars indicate 50 μm. Source data are provided as a Source Data file.

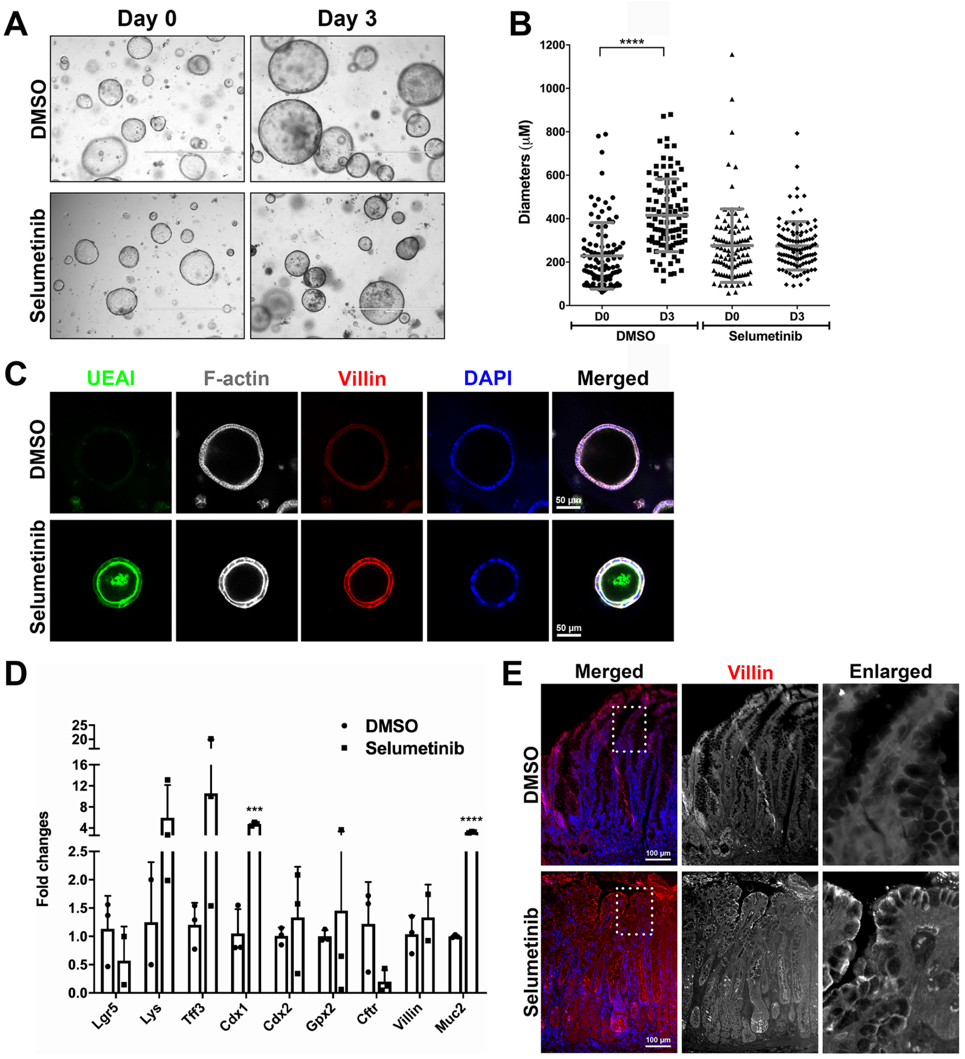

Supplementary Figure 7. Examination of cellular changes in Meta3 organoids after MEK inhibition. A) Meta3 organoids were treated with either DMSO containing control media or Selumetinib (1μM) containing media for 3 days. Phase contrast images were captured before and 3 days after the DMSO vehicle or Selumetinib treatment. B) Diameters of 100 Meta3 organoids were manually measured before and after either DMSO vehicle or Selumetinib treatment. Data are presented as mean values with standard deviation. P-values were calculated using unpaired two-tailed t-test. **** P<0.0001. C) Co-immunostaining for markers of enterocyte apical membrane, UEAI, villin and F-actin in paraffin embedded sections of Meta3 treated with either DMSO vehicle or Selumetinib. D) Expression of intestinal lineage marker transcripts after Selumetinib treatment. Quantitative PCR showing relative expression of intestinal lineage marker genes (*Lgr5, Lys, Tff3, Cdx1, Cdx2, Gpx2, Ctfr, Villin* and *Muc2*) 3 days after Selumetinib treatment. Data are presented as mean values with standard deviation (n=3). P-values were calculated using unpaired two-tailed t-test. *** P=0.0003 (*Cdx1*), **** P<0.0001 (*Muc2*). E) Immunostaining for Villin in the paraffin embedded Mist1-Kras stomach tissues treated with either DMSO vehicle or Selumetinib. Tissues were obtained from DMSO vehicle or Selumetinib (2 mg/kg) treated-Mist1-Kras mice at 3 months after tamoxifen injection.[1] Villin (red) was observed in the apical membrane of the remained metaplastic glands only after the Selumetinib treatment. Nuclei were counterstained with DAPI (grey). Dotted boxes indicate enlarged area. Source data are provided as a Source Data file.

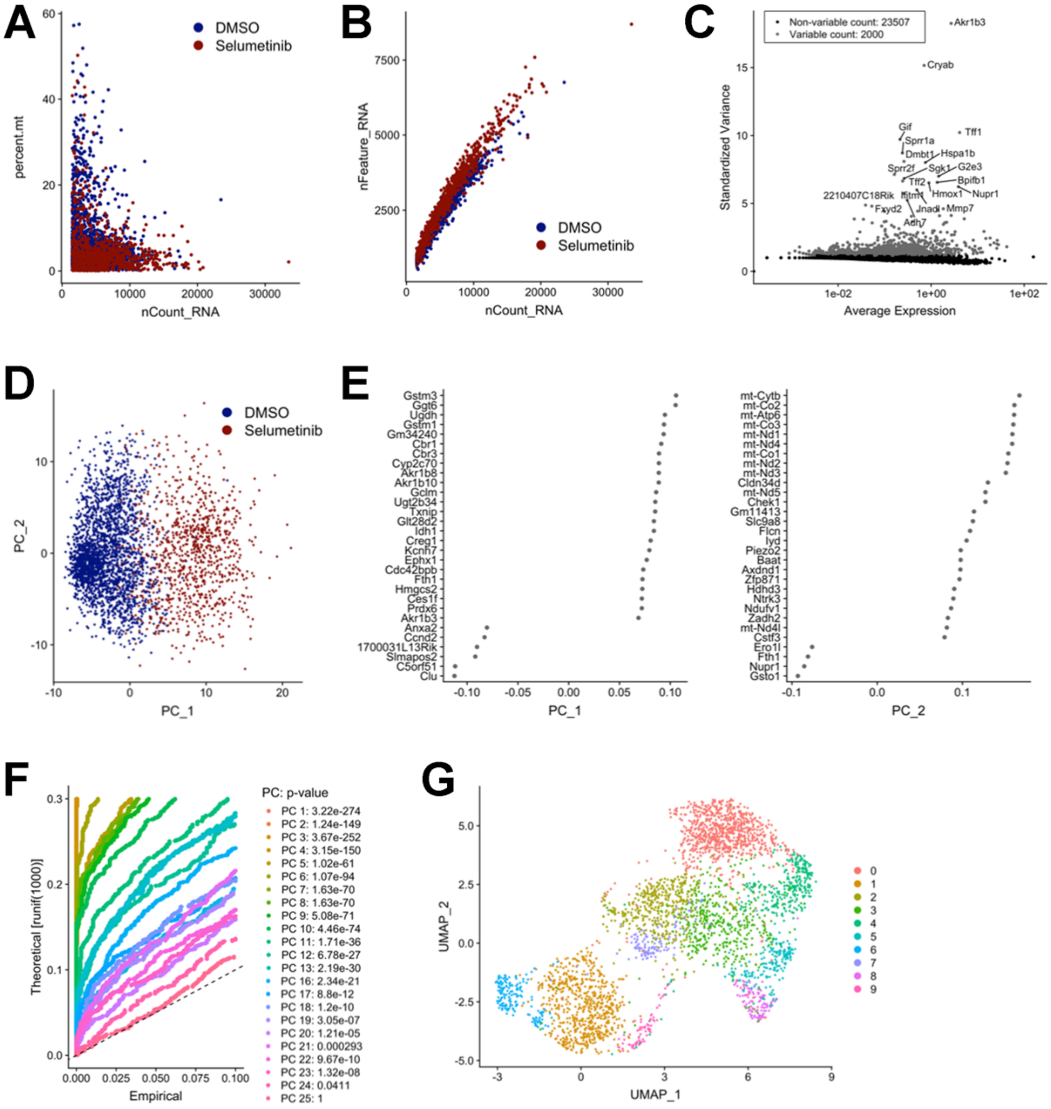

Supplementary Figure 8. Seurat pipeline used to analyze combined DMSO- and Selumetinib-treated Meta4 samples. A) percent mitochondrial expression (y-axis) versus number of counts/UMIs (x-axis) for DMSO vehicle-treated and Selumetinib-treated Meta4 samples. B) number of genes/features (y-axis) versus number of counts/UMIs (x-axis) for DMSO vehicle-treated and Selumetinib-treated Meta4 samples. C) Seurat's 'mean variability plot' showing highly variable genes (HVGs) for DMSO vehicle-treated and Selumetinib-treated Meta4 samples analyzed together. The top 20 most variable genes are labeled. D) Principle-component (PC) 2 versus 1 for DMSO vehicle-treated and Selumetinib-treated Meta4 samples analyzed together. E) Visualization of the top genes associated with the first 2 PCs for DMSO vehicle-treated and Selumetinib-treated Meta4 samples analyzed together. F) Plot of the JackStraw results used to determine the number of PCs to use for dimension reduction for DMSO vehicle-treated and Selumetinib-treated Meta4 samples analyzed together. G) UMAP dimension reduction results with Seurat clustering overlayed for DMSO vehicle-treated and Selumetinib-treated Meta4 samples analyzed and clustered together. Clustering results bias more clusters in the DMSO vehicle-treated sample and fail to sub-cluster Selumetinib-treated sample further.

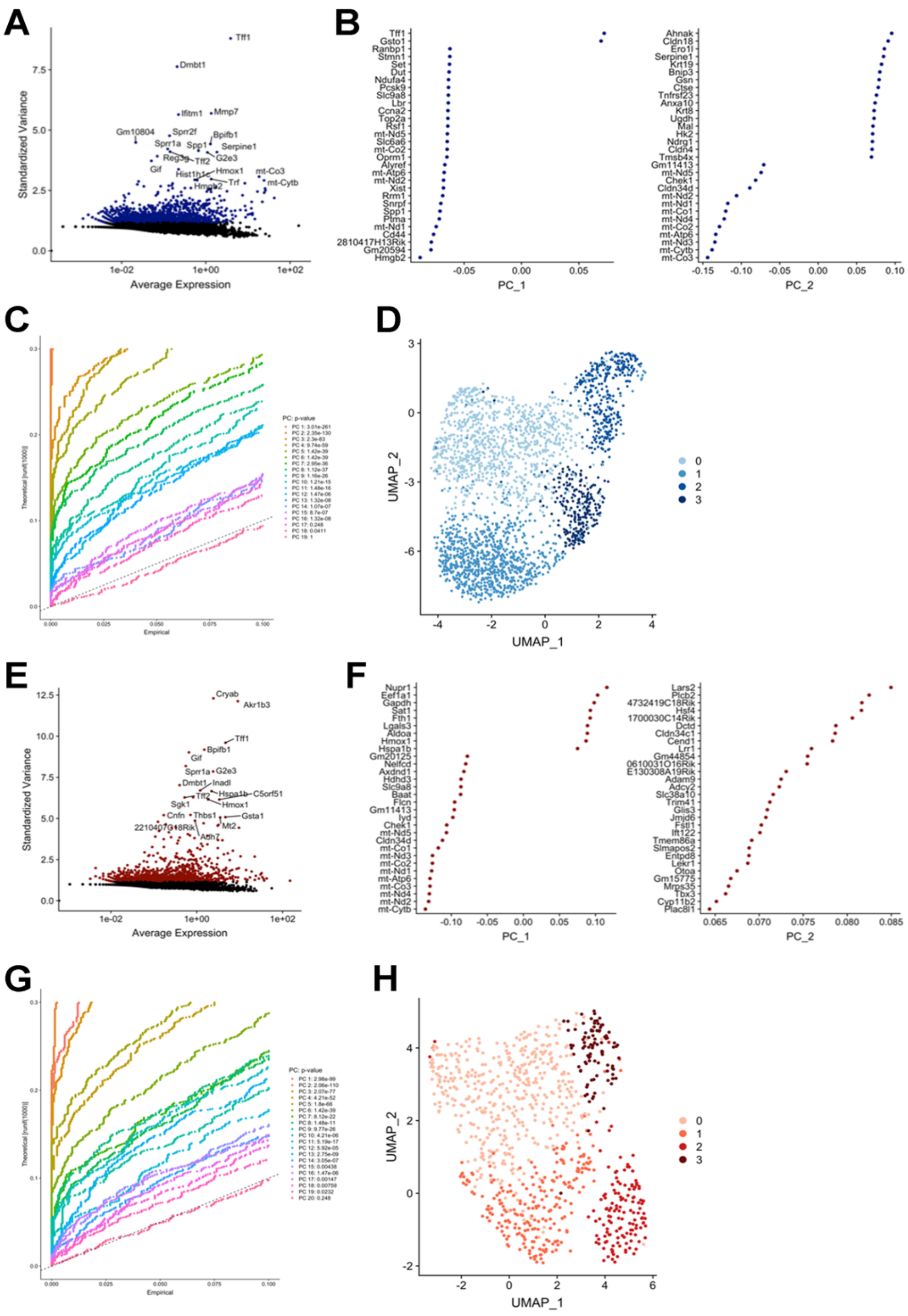

Supplementary Figure 9. Seurat pipeline used to analyze DMSO vehicle- and Selumetinib-treated Meta4 samples. A&E) Seurat's 'mean variability plot' showing highly variable genes (HVGs) for DMSO vehicle-treated (A) and Selumetinib-treated (E) Meta4 samples. The top 20 most variable genes are labeled for each sample. (B&F) Visualization of the top genes associated with the first 2 principle components (PCs) for DMSO vehicle-treated (B) and Selumetinib-treated (F) Meta4 samples. (C&G) Plot of the JackStraw results used to determine the number of PCs to use for dimension reduction for DMSO vehicle-treated (C) and Selumetinib-treated (G) Meta4 samples. (D&H) UMAP dimension reduction results with Seurat clustering overlayed for DMSO vehicle-treated (D) and Selumetinib-treated (H) Meta4 samples.

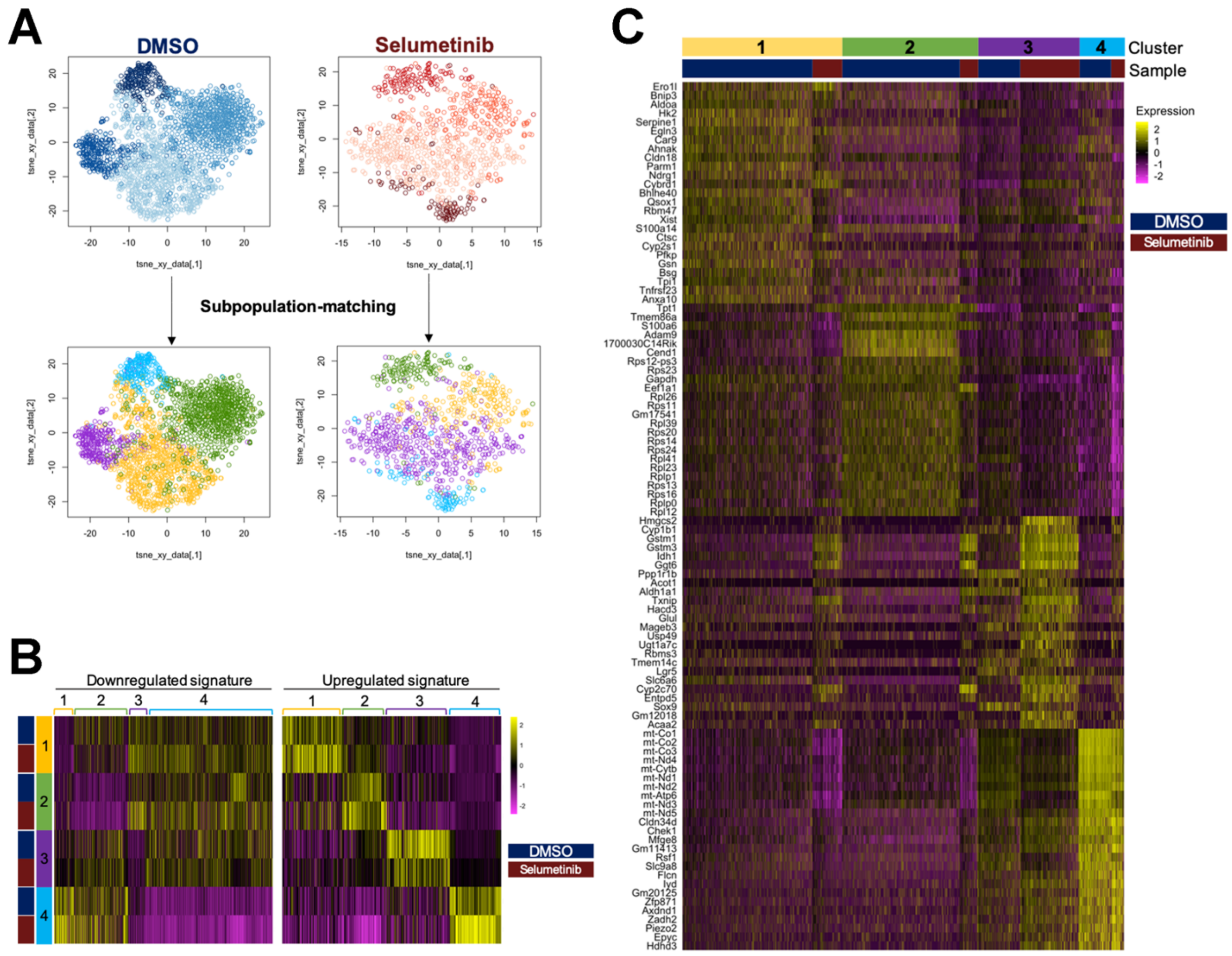

Supplementary Figure 10. Subpopulation-matching across Selumetinib treatment in Meta4 samples. A) t-SNE plots of DMSO vehicle-treated and Selumetinib-treated Meta4 samples with unmatched (top) and subpopulation-matched (bottom) clusters overlayed, represented by color. Clustering was performed on individual samples (DMSO vehicle- or Selumetinib-treated) using the Seurat pipeline. Subsequent subpopulation-matching was performed using an in-house algorithm that uses gene signatures of each cluster to identify similar cell types or states. B) Heatmaps showing the downregulated gene signatures from subpopulation-matching (left) and upregulated gene signatures from subpopulation-matching (right). Rows are organized by sample (DMSO vehicle-treated or Selumetinib-treated) as well as by matched-subpopulation. Columns are individual downregulated (left) or upregulated (right) genes arranged into gene signature groups for each cluster. The color indicates the average expression level for all cells within the cluster for an individual gene. Yellow corresponds to upregulation, black corresponds to neither upregulated or downregulated, and pink corresponds to downregulation. C) Heatmap of approximately 25 of the most upregulated genes in each subpopulation-matched cluster (1-4), separated by sample (DMSO vehicle-treated or Selumetinib-treated). Upregulated genes were defined as those expressed in at least 25% of the cells in the cluster with at least 0.25 log fold-change over all other cells. P-values were calculated using a two-tailed Wilcoxon Rank Sum test with Bonferroni correction and were < 1e-35. Rows correspond to individual genes and columns are individual cells, arranged by sample and subpopulation. Yellow corresponds to high expression, black corresponds to neither high nor low expression, and purple corresponds to low expression.

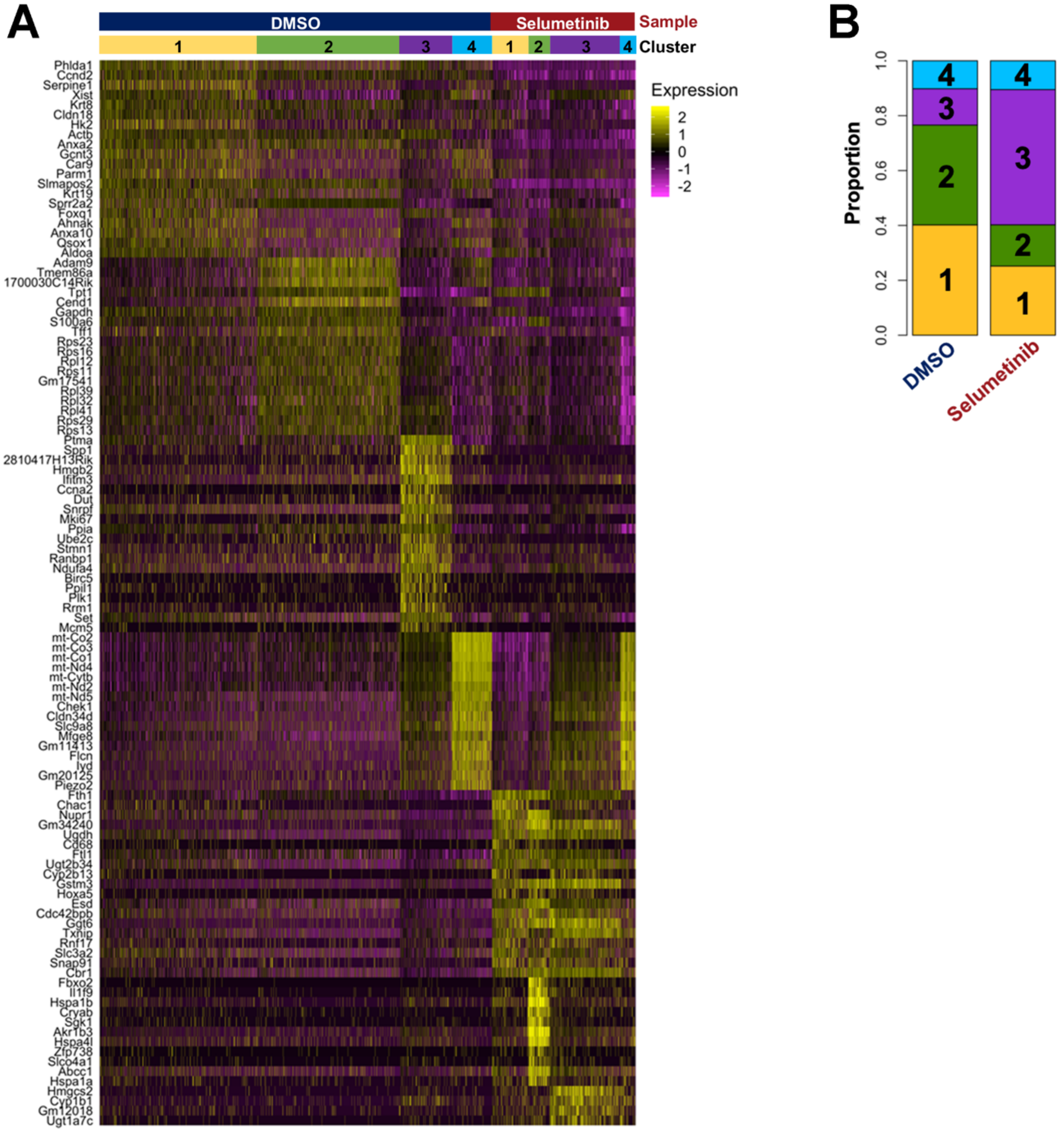

Supplementary Figure 11. Markers for subpopulation-matched clusters in DMSO vehicle-treated and Selumetinib-treated Meta4 samples. A) Heatmap of approximately 20 most upregulated genes in each subpopulation, specific to each sample (DMSO vehicle-treated or Selumetinib-treated). Upregulated genes were defined as those expressed in at least 25% of the cells in the cluster with at least 0.25 log fold-change over all other cells. P-values were calculated using a two-sided Wilcoxon Rank Sum test with Bonferroni correction and were < 1e-20. Rows correspond to individual genes and columns are individual cells, arranged by sample and subpopulation. Yellow corresponds to high expression, black corresponds to neither high nor low expression, and purple corresponds to low expression. B) Subpopulation proportions, based on number of cells, in the DMSO vehicle-treated and Selumetinib-treated Meta4 samples.

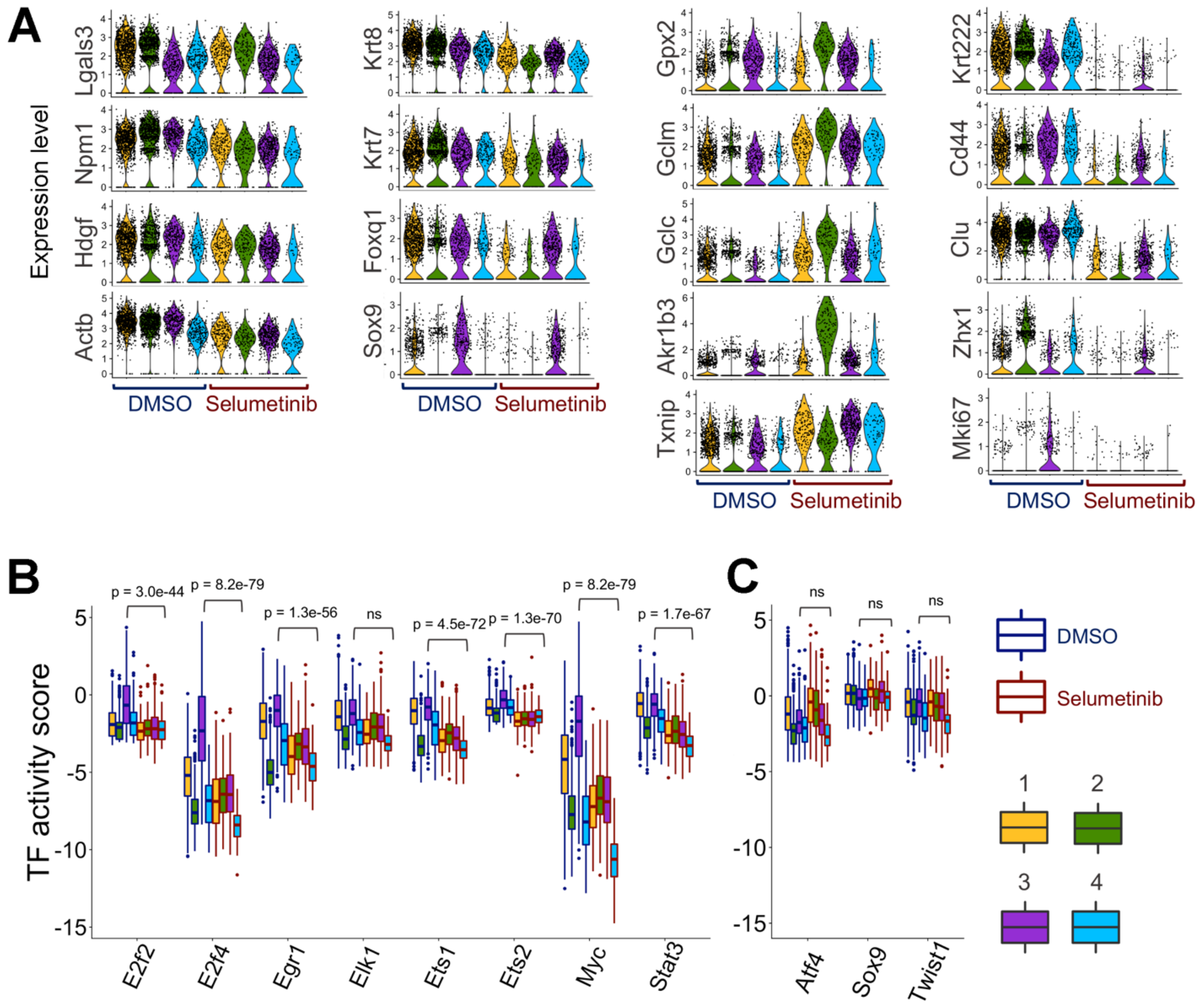

Supplementary Figure 12. Comparison of selected markers for matched subpopulations between DMSO vehicle- and Selumetinib-treated Meta4. A) Violin plots for relative expression of select genes. Results are shown for matched subpopulations (1-4) and split by treatment (DMSO vehicle or Selumetinib). B-C) Transcription factor (TF) activity scores, as inferred by the DoRothEA algorithm, for TFs involved in the MEK pathway (B) and not involved in the MEK pathway (C). Comparisons were made between DMSO-treated subpopulation 3 (n = 327 cells examined in one single-cell encapsulation) and Selumetinib-treated subpopulation 3 (n = 446 cells examined in one single-cell encapsulation) and statistically significant p-values are reported, as determined by unpaired, two-tailed Mann-Whitney tests with a 95% confidence level and adjusted using the Benjamini & Hochberg correction. The boxplot visualizes summary statistics including the median (center) and the 25th and 75th percentiles (bounds of the boxplot). The boxplot whiskers indicate values above and below 1.5x the inter-quartile range. All data points outside the inter-quartile range are outliers, but are not removed in statistical calculations.

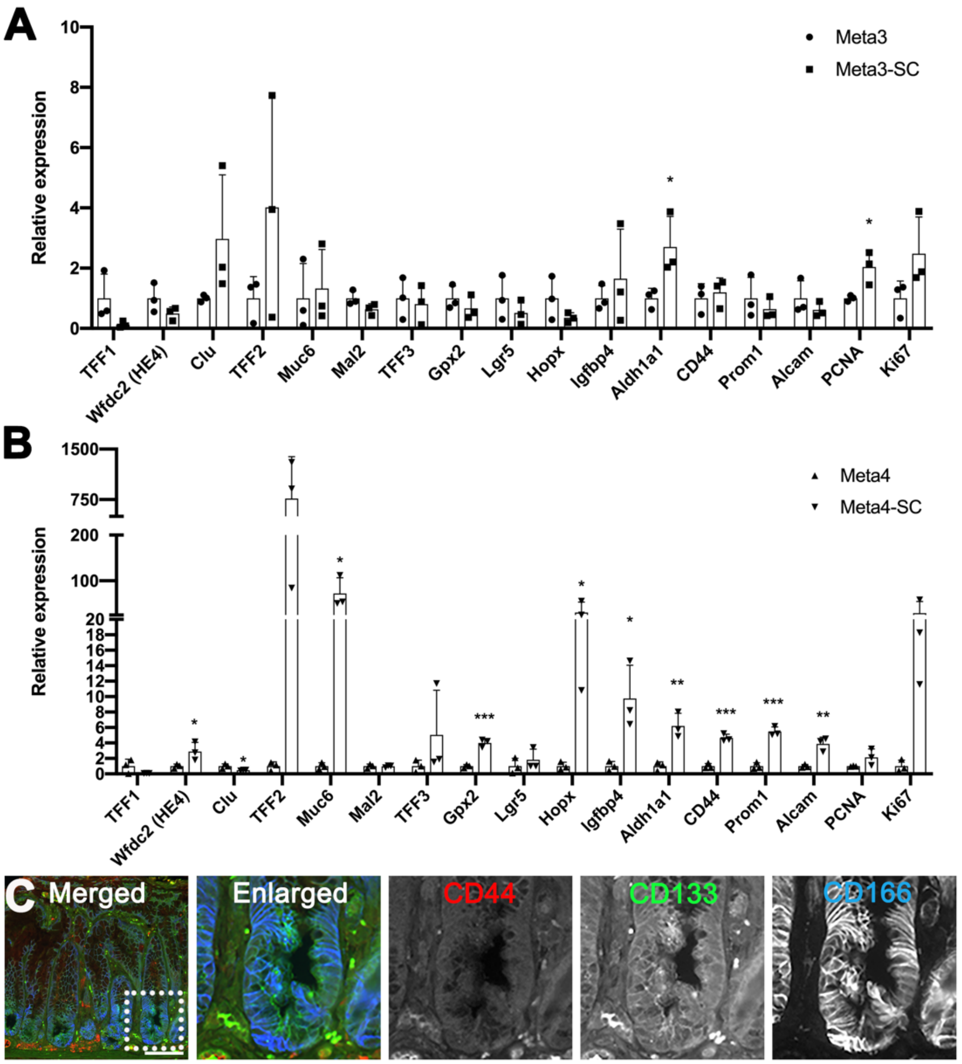

Supplementary Figure 13. Examination of stem cells in Meta3 and Meta4. A) Quantitative PCR showing relative expression levels of differentiated lineage markers (*TFF1, Wfdc2, Clu, TFF2, Muc6, Mal2, TFF3* and *Gpx2*), stem cell-related markers (*Lgr5, Hopx, Igfbp4, Aldh1a1, CD44, Prom1* and *Alcam*) and proliferation markers (*PCNA* and *Ki67*) between total Meta3 cells and sorted CD133+/CD166+ cells (SC) from Meta3. Expression levels were normalized to TBP gene. Data are presented as mean values with standard deviation (n=3). P-values were calculated using unpaired one-tailed t-test. * P<0.05 (0.03; *Aldh1a1*, 0.02;*PCNA*). B) Quantitative PCR showing relative expression levels of differentiated lineage markers (*TFF1, Wfdc2, Clu, TFF2, Muc6, Mal2, TFF3* and *Gpx2*), stem cell-related markers (*Lgr5, Hopx, Igfbp4, Aldh1a1, CD44, Prom1* and *Alcam*) and proliferation markers (*PCNA* and *Ki67*) between total Meta4 cells and sorted CD133+/CD166+ cells (SC) from Meta4. Expression levels were normalized to TBP gene. Data are presented as mean values with standard deviation (n=3). P-values were calculated using unpaired one-tailed t-test. * P<0.05 (0.02; *Clu*, 0.03; *HE4*, 0.01; *Muc6*, 0.04; *Hopx,* 0.01; *Igfbp4*), ** P<0.005 (0.003; *Aldh1a1*, 0.004; *Alcam*), *** P<0.0005 (0.0003; *Gpx1*, 0.0002; *CD44*, 0.0002; *Prom1*). C) Immunostaining for cancer stem cell markers, CD44, CD133 and CD166, in Mist1-Kras mice at 3 months after tamoxifen injection. Dotted box indicates enlarged area. Scale bar indicates 100 μm. Source data are provided as a Source Data file.

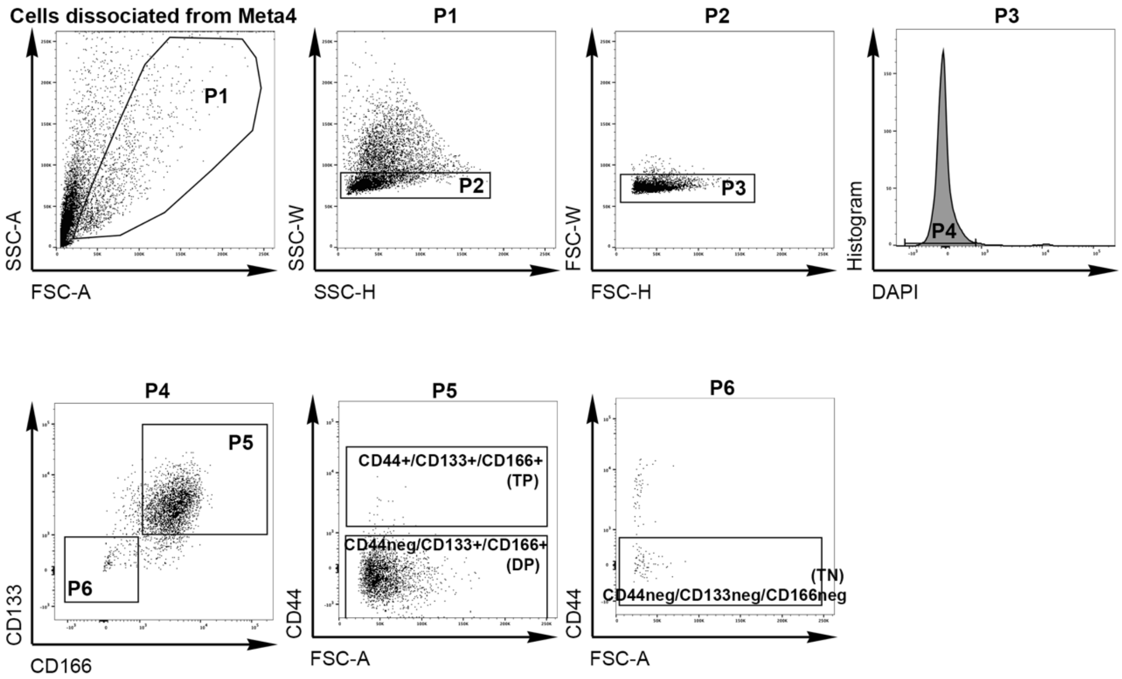

Supplementary Figure 14. Gating strategy to isolate CD44+/CD133+/CD166+ (TP), CD44neg/CD133+/CD166+ (DP) and CD44neg/CD133neg/CD166neg (TN) cells from Meta4 organoids (Fig 8A and 9C).

Supplementary Table 1. List of secondary antibodies used for immunofluorescence staining, reagents, primers, software and algorithms

Secondary antibody	Supplier	Catalog No	Dilutions	
Donkey anti-Rat IgG (H+L) Highly Cross-Adsorbed Secondary Antibody, Alexa Fluor 488	ThermoFisher	A-21208	1:500	N/A
Donkey anti-Rat IgG (H+L) Highly Cross-Adsorbed Secondary Antibody, Alexa Fluor 594	ThermoFisher	A-21209	1:500	N/A
Donkey anti-Rat IgG (H+L) Cross-Adsorbed Secondary Antibody, DyLight 680	ThermoFisher	SA5-10030	1:500	N/A
Donkey anti-Mouse IgG (H+L) Highly Cross-Adsorbed Secondary Antibody, Alexa Fluor 488	ThermoFisher	A-21202	1:500	N/A
Donkey anti-Mouse IgG (H+L) Highly Cross-Adsorbed Secondary Antibody, Alexa Fluor 555	ThermoFisher	A-31570	1:500	N/A
Donkey anti-Mouse IgG (H+L) Highly Cross-Adsorbed Secondary Antibody, Alexa Fluor 647	ThermoFisher	A-31571	1:500	N/A
Donkey anti-Rabbit IgG (H+L) Highly Cross-Adsorbed Secondary Antibody, Alexa Fluor 488	ThermoFisher	A-21206	1:500	N/A
Donkey anti-Rabbit IgG (H+L) Highly Cross-Adsorbed Secondary Antibody, Alexa Fluor 546	ThermoFisher	A10040	1:500	N/A
Donkey anti-Rabbit IgG (H+L) Highly Cross-Adsorbed Secondary Antibody, Alexa Fluor 647	ThermoFisher	A-31573	1:500	N/A

Reagents

Intesticult Organoid Growth Medium (mouse)	StemCell Technologies	6005
ECM Gel	Sigma-Aldrich	E1270
Penicillin Streptomycin Solution	Corning	30-002-CI
Amphotericin B solution	Sigma-Aldrich	A2942
MycoZap Plus PR	Lonza	VZA-2021
Y-27632 (ROCK inhibitor)	StemCell Technologies	72304
Collagen - PureCol Type 1	Advanced BioMatrix	5005

SytoxBlue	Thermo Fisher	S34857
AnnexinV-PacBlue	Biolegend	640918
iScript Reverse Transcription Supermix for RT-qPCR	Biorad	1708840
SsoAdvanced Universal SYBR Green Super Mix	Biorad	172-5270
**Primers**		
mLgr5 primers<br>F: ccaatggaataaagacgacggcaaca<br>R: gggccttcaggtcttcctcaaagtca	Sigma-Aldrich Genosys	N/A
mLysozyme (Lys) primers<br>F: atggaatggctggctactatgg<br>R: accagtatcggctattgatctga	Sigma-Aldrich Genosys	N/A
mTff3 primers<br>F: ttgctgggtcctctgggatag<br>R: tacactgctccgatgtgacag	Sigma-Aldrich Genosys	N/A
mCdx1 primers<br>F: ggacgccctacgaatgga<br>R: tctttacctgccgctctg	Sigma-Aldrich Genosys	N/A
mCdx2 primers<br>F: ctggagctggagaaggag<br>R: ggctctgcggttctgaaa	Sigma-Aldrich Genosys	N/A
mGpx2 primers<br>F: cagggctgtgctgattgag<br>R: cggacatacttgaggctgttc	Sigma-Aldrich Genosys	N/A
mCftr primers<br>F: ctggaccacaccaattttgagg<br>R: gcgtggataagctggggat	Sigma-Aldrich Genosys	N/A
mVillin primers<br>F: tcaaaggctctctcaacatcac<br>R: ggtgctggaaggaacagg	Sigma-Aldrich Genosys	N/A
mMuc2 primers<br>F: acaaaaaccccagcaacaag<br>R: gagcaagggactctggtctg	Sigma-Aldrich Genosys	N/A
mPanCD44 primers<br>F: acagtaccttacccaccatg<br>R: ggatgaatcctcggaatt	Sigma-Aldrich Genosys	N/A
mCD44v9 primers	Sigma-Aldrich Genosys	N/A

F: ggagatcaggatgactccttct		
R: agtccttggatgagtctcgatc		
mClusterin (Clu) primers	Sigma-Aldrich Genosys	N/A
F: ccagcctttctttgagatga		
R: ctcctggcacttttcacact		
mTff1 primers	Sigma-Aldrich Genosys	N/A
F: agcacaaggtgatctgtgtcc		
R: gaagccacaatttatcctctccc		
mAldh1 primers	Sigma-Aldrich Genosys	N/A
F: ccgtggcgtactatggatgc		
R: gcagcagacgatctctttcgat		
mIgfbp4 primers	Sigma-Aldrich Genosys	N/A
F: cggagcaagatgaagatcgtgg		
R: gatgaagaggtcttcgtgggtac		
mMal2 primers	Sigma-Aldrich Genosys	N/A
F: gctttcgtctgtctggagattg		
R: acacaaacatgacccatccttg		
mHopx primers	Sigma-Aldrich Genosys	N/A
F: tctccatccttagtcagacgc		
R: gggtgcttgttgaccttgtt		
mMuc6 primers	Sigma-Aldrich Genosys	N/A
F: ggaactaacagtctggaccacc		
R: cttcggtatggatgtaggaggc		
mTff2 primers	Sigma-Aldrich Genosys	N/A
F: tgctttgatcttggatgctg		
R: ggaaaagcagcagtttcgac		
mHE4 primers	Sigma-Aldrich Genosys	N/A
F: tgcctgcctgtcgcctctg		
R: tgtccgcacagtccttgtcca		
mMki67 primers	Sigma-Aldrich Genosys	N/A
F: atcattgaccgctcctttaggt		
R: gctcgccttgatggttcct		
mPcna primers	Sigma-Aldrich Genosys	N/A
F: gcgtgaacctcaccagtatgt		
R: tcttcggcccttagtgtaatgat		
mTBP primers	Sigma-Aldrich Genosys	N/A
F: caaacccagaattgttctcctt		

R: atgtggtcttcctgaatccct		
**Software and Algorithms**		
Zeiss ZEN	Zeiss	N/A
ImageJ		N/A
GraphPad Prism 7	GraphPad Software	N/A
JuLi™ Stage	Nanoentek	N/A
CFX Maestro	Bio-Rad	N/A
GelCount™	Oxford Optronix	N/A
FlowJo	BD	N/A
AxioVision SE64 Rel. 4.9.1	Zeiss	N/A

Supplementary Table 2. Software and Algorithms for scRNA-seq Analysis

Resource	Supplementary Reference
RStudio version 3.5.2	1
Seurat version 3.0.0	2
ggplot2 version 3.1.1	3
Picante version 1.8	4
Rtsne version 0.15	5
pheatmap version 1.0.12	6
abind version 1.4-5	7
RColorBrewer version 1.1-2	8
Cowplot version 0.9.4	9
Ggthemes version 4.2.0	10
Dplyr version 0.8.1	11
reshape2 version 1.4.3	12
Viper version 1.16.0	13
inDrops pipeline	14
Python version 3.7	15
Numpy version 1.15.4	16
Pandas version 0.23.4	17
Argparse version 1.1	18
Scipy version 1.1.0	19
Matplotlib version 3.0.2	20
Seaborn version 0.9.0	21
Umap version 0.3.8	22

Supplementary References

1. R Core Team (2018). R: A language and environment for statistical computing. R Foundation for Statistical Computing, Vienna, Austria. URL https://www.R-project.org/.
2. Stuart and Butler et al. Comprehensive integration of single cell data. bioRxiv (2018).
3. H. Wickham. ggplot2: Elegant Graphics for Data Analysis. Springer-Verlag New York, 2016.
4. S.W. Kembel, P.D. Cowan, M.R. Helmus, W.K. Cornwell, H. Morlon, D.D. Ackerly, S.P. Blomberg, and C.O. Webb. 2010. Picante: R tools for integrating phylogenies and ecology. Bioinformatics 26:1463-1464.
5. Jesse H. Krijthe (2015). Rtsne: T-Distributed Stochastic Neighbor Embedding using a Barnes-Hut Implementation, URL: https://github.com/jkrijthe/Rtsne
6. Raivo Kolde (2019). pheatmap: Pretty Heatmaps. R package version 1.0.12. https://CRAN.R-project.org/package=pheatmap
7. Tony Plate and Richard Heiberger (2016). abind: Combine Multidimensional Arrays. R package version 1.4-5. https://CRAN.R-project.org/package=abind
8. Erich Neuwirth (2014). RColorBrewer: ColorBrewer Palettes. R package version 1.1-2. https://CRAN.R-project.org/package=RColorBrewer
9. Claus O. Wilke (2019). cowplot: Streamlined Plot Theme and Plot Annotations for 'ggplot2'. R package version 0.9.4. https://CRAN.R-project.org/package=cowplot
10. Jeffrey B. Arnold (2019). ggthemes: Extra Themes, Scales and Geoms for 'ggplot2'. R package version 4.2.0. https://CRAN.R-project.org/package=ggthemes
11. Hadley Wickham, Romain François, Lionel Henry and Kirill Müller (2019). dplyr: A Grammar of Data Manipulation. R package version 0.8.1. https://CRAN.R-project.org/package=dplyr
12. Hadley Wickham (2007). Reshaping Data with the reshape Package. Journal of Statistical Software, 21(12), 1-20. URL http://www.jstatsoft.org/v21/i12/.
13. Alvarez, M.J., Shen, Y., Giorgi, F.M., Lachmann, A., Ding, B.B., Ye, B.H., and Califano, A. (2016) Functional characterization of somatic mutations in cancer using network-based inference of protein activity. Nature genetics 48(8):848--47.
14. Klein, A. M., Mazutis, L., Akartuna, I., Tallapragada, N., Veres, A., Li, V., ... & Kirschner, M. W. (2015). Droplet barcoding for single-cell transcriptomics applied to embryonic stem cells. *Cell*, *161*(5), 1187-1201.
15. Van Rossum, G., & Drake Jr, F. L. (1995). *Python tutorial*. Centrum voor Wiskunde en Informatica Amsterdam, The Netherlands.
16. Oliphant, T. E. (2006). *A guide to NumPy* (Vol. 1). Trelgol Publishing USA.
17. McKinney, W., & others. (2010). Data structures for statistical computing in python. In *Proceedings of the 9th Python in Science Conference* (Vol. 445, pp. 51–56).
18. https://github.com/python/cpython/blob/master/Lib/argparse.py
19. Jones, E., Oliphant, T., Peterson, P., & others. (2001). *SciPy: Open source scientific tools for Python*. Retrieved from "http://www.scipy.org/"
20. Hunter, J. D. (2007). Matplotlib: A 2D graphics environment. *Computing in Science & Engineering*, *9*(3), 90–95.
21. https://github.com/mwaskom/seaborn
22. McInnes, L, Healy, J, *UMAP: Uniform Manifold Approximation and Projection for Dimension Reduction*, ArXiv e-prints 1802.03426, 2018
