# Supplementary material for: Heterogeneity and dynamics of active Kras-induced dysplastic lineages from mouse corpus stomach
Source: Nat Commun. 2019 Dec 5;10:5549. doi: 10.1038/s41467-019-13479-6 (PMC6895174; doi:10.1038/s41467-019-13479-6)
Supplement: Supplementary file 5 — Reporting Summary [file 41467_2019_13479_MOESM5_ESM.pdf]

nature research

Corresponding author(s): Eunyoung Choi, Ph.D.

Last updated by author(s): Oct 28, 2019

Reporting Summary

Nature Research wishes to improve the reproducibility of the work that we publish. This form provides structure for consistency and transparency in reporting. For further information on Nature Research policies, see Authors & Referees and the Editorial Policy Checklist .

Statistics

For all statistical analyses, confirm that the following items are present in the figure legend, table legend, main text, or Methods section.

n/a	Confirmed	
☐	☒	The exact sample size (*n*) for each experimental group/condition, given as a discrete number and unit of measurement
☐	☒	A statement on whether measurements were taken from distinct samples or whether the same sample was measured repeatedly
☐	☒	The statistical test(s) used AND whether they are one- or two-sided<br>*Only common tests should be described solely by name; describe more complex techniques in the Methods section.*
☐	☒	A description of all covariates tested
☐	☒	A description of any assumptions or corrections, such as tests of normality and adjustment for multiple comparisons
☐	☒	A full description of the statistical parameters including central tendency (e.g. means) or other basic estimates (e.g. regression coefficient) AND variation (e.g. standard deviation) or associated estimates of uncertainty (e.g. confidence intervals)
☐	☒	For null hypothesis testing, the test statistic (e.g. *F*, *t*, *r*) with confidence intervals, effect sizes, degrees of freedom and *P* value noted<br>*Give P values as exact values whenever suitable.*
☒	☐	For Bayesian analysis, information on the choice of priors and Markov chain Monte Carlo settings
☒	☐	For hierarchical and complex designs, identification of the appropriate level for tests and full reporting of outcomes
☒	☐	Estimates of effect sizes (e.g. Cohen's *d*, Pearson's *r*), indicating how they were calculated

Our web collection on statistics for biologists contains articles on many of the points above.

Software and code

Policy information about availability of computer code

Data collection	Prism 8, GelCount, CFX Maestro, ZEISS ZEN, JuLI Stage,
Data analysis	Prism 8, GelCount, CFX Maestro, DoRothEA package v2, JuLI Stage, ZEISS ZEN, AxioVision SE64 Rel. 4.9.1, RStudio 3.5.2, Seurat 3.0.0, ggplot2 3.1.1, Picante 1.8, Rtsne 0.15, pheatmap 1.0.12, abind 1.4-5, RColorBrewer 1.1-2, Cowplot 0.9.4, Ggthemes 4.2.0, Dplyr 0.8.1, reshape2 1.4.3, Viper 1.16.0, inDrops pipeline, Python 3.7, Numpy 1.15.4, Pandas 0.23.4, Argparse 1.1, Scipy 1.1.0, Matplotlib 3.0.2, Seaborn 0.9.0, Umap 0.3.8

For manuscripts utilizing custom algorithms or software that are central to the research but not yet described in published literature, software must be made available to editors/reviewers. We strongly encourage code deposition in a community repository (e.g. GitHub). See the Nature Research guidelines for submitting code & software for further information.

Data

Policy information about availability of data

All manuscripts must include a data availability statement. This statement should provide the following information, where applicable:

- Accession codes, unique identifiers, or web links for publicly available datasets
- A list of figures that have associated raw data
- A description of any restrictions on data availability

Two scRNA-seq data reported in figure 2, 6 and 7, have been submitted to NCBI Gene Expression Omnibus (GEO): GSE121940 and all the data sets have been released publicly.

Field-specific reporting

Please select the one below that is the best fit for your research. If you are not sure, read the appropriate sections before making your selection.

- [x] Life sciences
- [ ] Behavioural & social sciences
- [ ] Ecological, evolutionary & environmental sciences

For a reference copy of the document with all sections, see nature.com/documents/nr-reporting-summary-flat.pdf

Life sciences study design

All studies must disclose on these points even when the disclosure is negative.

Sample size	We performed at least 3 independent experiments and capture multiple images on multiple tissues or wells. We specified the number of cells and independent experiments in the figure legends and/or methods. Fifteen to twenty wells of Meta4 organoids per condition were used for FACS analysis. A total of 5,000 cells per group were used for clonality analysis using CRA microwell system. About 50 organoids were used for in vitro assays. A total of 50 to 100 organoids were considered from three representative images taken from three wells of each organoid line for quantitative analyses.
Data exclusions	We only exclude analyses if there is an obvious reason for poor data, such as dead or sick-looking organoids prior to drug treatment.
Replication	All attempts at replication are successful. We established 3 metaplastic and 3 dysplastic organoids lines from independent mouse stomach tissues. We performed at least 3 independent experiments and capture multiple images on multiple tissues or wells.
Randomization	Both male and female mice were randomly used to establish organoid lines.
Blinding	The mouse alleles which were used for this study are C57BL/6 wild-type or Kras-induced mice to establish metaplastic or dysplastic organoid lines. Thus, investigators were not blinded to mouse genotypes during experiments.

Reporting for specific materials, systems and methods

We require information from authors about some types of materials, experimental systems and methods used in many studies. Here, indicate whether each material, system or method listed is relevant to your study. If you are not sure if a list item applies to your research, read the appropriate section before selecting a response.

Materials & experimental systems

n/a	Involved in the study	
[ ]	[x]	Antibodies
[ ]	[x]	Eukaryotic cell lines
[x]	[ ]	Palaeontology
[ ]	[x]	Animals and other organisms
[x]	[ ]	Human research participants
[x]	[ ]	Clinical data

Methods

n/a	Involved in the study	
[x]	[ ]	ChIP-seq
[ ]	[x]	Flow cytometry
[x]	[ ]	MRI-based neuroimaging

Antibodies

Antibodies used

Anti CD44 Antigen v10-e16 mAb (Clone RM1, CD44v9), Cosmo Bio CAC-LKG-M002, 1:25,000, Rat
Lectin from Ulex Europaeus (UEA1-lectin), Sigma L9006, 1:2,000
CDX1 Polyclonal Antibody, Thermo Scientific PA5-23056, 1:500, Rabbit
Anti-Sox9 Polyclonal Antibody, Millipore Ab5535, 1:1,500, Rabbit
Alexa Fluor® 700 anti-human CD44 Antibody (Clone BJ18), Biolegend 338813, 5 µl per million cells in 100 µl staining volume, Mouse
Alexa Fluor® 647 anti-mouse CD133 Antibody (Clone 315-2C11), Biolegend 141215, 0.5 mg/ml, Rat
PE anti-mouse CD133 Antibody (Clone 315-2C11), Biolegend 141204, 0.2 mg/ml, Rat
Mouse ALCAM/CD166 PE-conjugated Antibody (Polyclonal), R&D systems FAB1172P, 10 µL/10^6 cells, Goat
Mouse ALCAM/CD166 Fluorescein-conjugated Antibody (Polyclonal), R&D systems FAB1172F, 10 µL/10^6 cells, Goat
CD44 Monoclonal Antibody (8E2F3), Invitrogen MA5-15462, 1:1,000, Mouse
CD133 (Prominin-1) Monoclonal Antibody (13A4), eBioscience 14-1331-80, 1:100, Rat
Recombinant Anti-CD166 antibody [EPR2759(2)], Abcam ab109215, 1:250, Rabbit
Purified anti-mouse Ki-67 Antibody (Clone 16A8), Biolegend 652402, 1:200, Rat
Villin-1 (R814) Antibody, Cell Signaling Technology 2369, 1:300, Rabbit
Monoclonal Anti-Vimentin antibody produced in mouse (Clone V9), Sigma-Aldrich V6630, 1:300, Mouse
Pan-cytokeratin (polyclonal), Dako Z0622, 1:4,000, Rabbit
Phospho-Ezrin (Thr567)/Radixin (Thr564)/Moesin (Thr558) (48G2), Cell Signaling Technology 3726, 1:300, Rabbit

Anti-Cortactin (p80/85) Antibody, clone 4F11, Millipore 05-180, 1:1,000, Mouse
Alexa Fluor™ 647 Phalloidin, Invitrogen A22287, 1:100
p44/42 MAPK (Erk1/2) (137F5), Cell Signaling Technology 4695, 1:1,000, Rabbit
Phospho-p44/42 MAPK (Erk1/2) (Thr202/Tyr204) (D13.14.4E) XP®, Cell Signaling Technology 4370S, 1:2,000, Rabbit
Monoclonal Anti-β-Actin antibody produced in mouse, Sigma A5316, 1:2,500, Mouse
RFP Antibody Pre-adsorbed (Polyclonal, tdTOM), Rockland 600-401-379, 1:500, Rabbit
Donkey anti-Rat IgG (H+L) Highly Cross-Adsorbed Secondary Antibody, Alexa Fluor 488, Invitrogen A-21208, 1:500
Donkey anti-Rat IgG (H+L) Highly Cross-Adsorbed Secondary Antibody, Alexa Fluor 594, Invitrogen A-21209, 1:500
Donkey anti-Rat IgG (H+L) Cross-Adsorbed Secondary Antibody, DyLight 680, Invitrogen SA5-10030, 1:500
Donkey anti-Mouse IgG (H+L) Highly Cross-Adsorbed Secondary Antibody, Alexa Fluor 488, Invitrogen A-21202, 1:500
Donkey anti-Mouse IgG (H+L) Highly Cross-Adsorbed Secondary Antibody, Alexa Fluor 555, Invitrogen A-31570, 1:500
Donkey anti-Mouse IgG (H+L) Highly Cross-Adsorbed Secondary Antibody, Alexa Fluor 647, Invitrogen A-31571, 1:500
Donkey anti-Rabbit IgG (H+L) Highly Cross-Adsorbed Secondary Antibody, Alexa Fluor 488, Invitrogen A-21206, 1:500
Donkey anti-Rabbit IgG (H+L) Highly Cross-Adsorbed Secondary Antibody, Alexa Fluor 546, Invitrogen A10040, 1:500
Donkey anti-Rabbit IgG (H+L) Highly Cross-Adsorbed Secondary Antibody, Alexa Fluor 647, Invitrogen A-31573, 1:500
IRDye® 800CW Donkey anti-Rabbit IgG Secondary Antibody LI-COR 926-32213 1:15,000
IRDye® 680LT Donkey anti-Mouse IgG Secondary Antibody LI-COR 926-68022 1:15,000

Validation

Anti CD44 Antigen v10-e16 mAb (Clone RM1, CD44v9), Cosmo Bio CAC-LKG-M002, https://www.cosmobiousa.com/products/anti-cd44-v10-e16-mab-clone-rm1
Lectin from Ulex Europaeus (UEA1-lectin), Sigma L9006, https://www.sigmaaldrich.com/catalog/product/sigma/l8146?lang=en&region=US
CDX1 Polyclonal Antibody, Thermo Scientific PA5-23056, https://www.thermofisher.com/antibody/product/CDX1-Antibody-Polyclonal/PA5-23056
Anti-Sox9 Polyclonal Antibody, Millipore Ab5535, http://www.emdmillipore.com/US/en/product/Anti-Sox9-Antibody,MM_NF-AB5535
Alexa Fluor® 700 anti-human CD44 Antibody (Clone BJ18), Biolegend 338813, https://www.biolegend.com/en-us/products/alexa-fluor-700-anti-human-cd44-antibody-14814
Alexa Fluor® 647 anti-mouse CD133 Antibody (Clone 315-2C11), Biolegend 141215, https://www.biolegend.com/nl-nl/products/alexa-fluor-647-anti-mouse-cd133-antibody-12188
PE anti-mouse CD133 Antibody (Clone 315-2C11), Biolegend 141204, https://www.biolegend.com/en-us/products/pe-anti-mouse-cd133-antibody-7066
Mouse ALCAM/CD166 PE-conjugated Antibody (Polyclonal), R&D systems FAB1172P, https://www.rndsystems.com/products/mouse-alcam-cd166-pe-conjugated-antibody_fab1172p
Mouse ALCAM/CD166 Fluorescein-conjugated Antibody (Polyclonal), R&D systems FAB1172F, https://www.rndsystems.com/products/mouse-alcam-cd166-fluorescein-conjugated-antibody_fab1172f
CD44 Monoclonal Antibody (8E2F3), Invitrogen MA5-15462, https://www.thermofisher.com/antibody/product/CD44-Antibody-clone-8E2F3-Monoclonal/MA5-15462
CD133 (Prominin-1) Monoclonal Antibody (13A4), eBioscience 14-1331-80, https://www.thermofisher.com/antibody/product/CD133-Prominin-1-Antibody-clone-13A4-Monoclonal/14-1331-82
Recombinant Anti-CD166 antibody [EPR2759(2)], Abcam ab109215, https://www.abcam.com/cd166-antibody-epr27592-ab109215.html
Purified anti-mouse Ki-67 Antibody (Clone 16A8), Biolegend 652402, https://www.biolegend.com/en-us/products/purified-anti-mouse-ki-67-antibody-7840
Villin-1 (R814) Antibody, Cell Signaling Technology 2369, https://www.cellsignal.com/products/primary-antibodies/villin-1-r814-antibody/2369
Monoclonal Anti-Vimentin antibody produced in mouse (Clone V9), Sigma-Aldrich V6630, https://www.sigmaaldrich.com/catalog/product/sigma/v6630?lang=en&region=US
Pan-cytokeratin (polyclonal), Dako Z0622, https://www.labome.com/product/Dako/Z0622.html
Phospho-Ezrin (Thr567)/Radixin (Thr564)/Moesin (Thr558) (48G2), Cell Signaling Technology 3726, https://www.cellsignal.com/products/primary-antibodies/phospho-ezrin-thr567-radixin-thr564-moesin-thr558-48g2-rabbit-mab/3726
Anti-Cortactin (p80/85) Antibody, clone 4F11, Millipore 05-180, http://www.emdmillipore.com/US/en/product/Anti-Cortactin-p80-85-Antibody-clone-4F11,MM_NF-05-180
Alexa Fluor™ 647 Phalloidin, Invitrogen A22287, https://www.thermofisher.com/order/catalog/product/A22287
p44/42 MAPK (Erk1/2) (137F5), Cell Signaling Technology 4695, https://www.cellsignal.com/products/primary-antibodies/p44-42-mapk-erk1-2-137f5-rabbit-mab/4695
Phospho-p44/42 MAPK (Erk1/2) (Thr202/Tyr204) (D13.14.4E) XP®, Cell Signaling Technology 4370S, https://www.cellsignal.com/products/primary-antibodies/phospho-p44-42-mapk-erk1-2-thr202-tyr204-d13-14-4e-xp-rabbit-mab/4370
Monoclonal Anti-β-Actin antibody produced in mouse, Sigma A5316, https://www.sigmaaldrich.com/catalog/product/sigma/a5316?lang=en&region=US
RFP Antibody Pre-adsorbed (Polyclonal, tdTOM), Rockland 600-401-379, https://rockland-inc.com/store/Antibodies-to-GFP-and-Antibodies-to-RFP-600-401-379-O4L_24299.aspx
Donkey anti-Rat IgG (H+L) Highly Cross-Adsorbed Secondary Antibody, Alexa Fluor 488, Invitrogen A-21208, https://www.thermofisher.com/antibody/product/Donkey-anti-Rat-IgG-H-L-Highly-Cross-Adsorbed-Secondary-Antibody-Polyclonal/A-21208
Donkey anti-Rat IgG (H+L) Highly Cross-Adsorbed Secondary Antibody, Alexa Fluor 594, Invitrogen A-21209, https://www.thermofisher.com/antibody/product/Donkey-anti-Rat-IgG-H-L-Highly-Cross-Adsorbed-Secondary-Antibody-Polyclonal/A-21209

Donkey anti-Rat IgG (H+L) Cross-Adsorbed Secondary Antibody, DyLight 680, Invitrogen SA5-10030, https://www.thermofisher.com/antibody/product/Donkey-anti-Rat-IgG-H-L-Cross-Adsorbed-Secondary-Antibody-Polyclonal/SA5-10030
Donkey anti-Mouse IgG (H+L) Highly Cross-Adsorbed Secondary Antibody, Alexa Fluor 488, Invitrogen A-21202, https://www.thermofisher.com/antibody/product/Donkey-anti-Mouse-IgG-H-L-Highly-Cross-Adsorbed-Secondary-Antibody-Polyclonal/A-21202
Donkey anti-Mouse IgG (H+L) Highly Cross-Adsorbed Secondary Antibody, Alexa Fluor 555, Invitrogen A-31570, https://www.thermofisher.com/antibody/product/Donkey-anti-Mouse-IgG-H-L-Highly-Cross-Adsorbed-Secondary-Antibody-Polyclonal/A-31570
Donkey anti-Mouse IgG (H+L) Highly Cross-Adsorbed Secondary Antibody, Alexa Fluor 647, Invitrogen A-31571, https://www.thermofisher.com/antibody/product/Donkey-anti-Mouse-IgG-H-L-Highly-Cross-Adsorbed-Secondary-Antibody-Polyclonal/A-31571
Donkey anti-Rabbit IgG (H+L) Highly Cross-Adsorbed Secondary Antibody, Alexa Fluor 488, Invitrogen A-21206, https://www.thermofisher.com/antibody/product/Donkey-anti-Rabbit-IgG-H-L-Highly-Cross-Adsorbed-Secondary-Antibody-Polyclonal/A-21206
Donkey anti-Rabbit IgG (H+L) Highly Cross-Adsorbed Secondary Antibody, Alexa Fluor 546, Invitrogen A10040, https://www.thermofisher.com/antibody/product/Donkey-anti-Rabbit-IgG-H-L-Highly-Cross-Adsorbed-Secondary-Antibody-Polyclonal/A10040
Donkey anti-Rabbit IgG (H+L) Highly Cross-Adsorbed Secondary Antibody, Alexa Fluor 647, Invitrogen A-31573, https://www.thermofisher.com/antibody/product/Donkey-anti-Rabbit-IgG-H-L-Highly-Cross-Adsorbed-Secondary-Antibody-Polyclonal/A-31573
IRDye® 800CW Donkey anti-Rabbit IgG Secondary Antibody, LI-COR 926-32213, https://www.licor.com/bio/reagents/irdye-800cw-donkey-anti-rabbit-igg-secondary-antibody
IRDye® 680LT Donkey anti-Mouse IgG Secondary Antibody, LI-COR 926-68022, https://www.licor.com/bio/reagents/irdye-680lt-donkey-anti-mouse-igg-secondary-antibody

Eukaryotic cell lines

Policy information about cell lines

Cell line source(s)	Novel organoid lines established from mouse stomach tissues
Authentication	None of the cell lines were authenticated.
Mycoplasma contamination	All organoid lines were tested negative for mycoplasma contamination and culture media contain Mycoplasma elimination reagent.
Commonly misidentified lines (See ICLAC register)	None

Animals and other organisms

Policy information about studies involving animals; ARRIVE guidelines recommended for reporting animal research

Laboratory animals	6-8 weeks old C57BL/6 mice. Both male and female mice were used.
Wild animals	This study did not involve wild animals.
Field-collected samples	This study did not involve samples collected from the field.
Ethics oversight	All mouse imaging and surgical protocols were approved by the Institutional Animal Care and Use Committee of Vanderbilt University.

Note that full information on the approval of the study protocol must also be provided in the manuscript.

Flow Cytometry

Plots

Confirm that:

- [x] The axis labels state the marker and fluorochrome used (e.g. CD4-FITC).
- [x] The axis scales are clearly visible. Include numbers along axes only for bottom left plot of group (a 'group' is an analysis of identical markers).
- [x] All plots are contour plots with outliers or pseudocolor plots.
- [x] A numerical value for number of cells or percentage (with statistics) is provided.

Methodology

Sample preparation	Meta4 cells were isolated from the Meta4 organoids as described in the Supplemental Methods section.

Instrument	FACSAria III & LSR II flow cytometer (BD Biosciences), and Sony SH800
Software	BD FACSDiva software or SH800S Cell Sorter was used for data collection and FlowJo v10 for data analysis.
Cell population abundance	Live cells were typically over 85% of single cells, and CD133+/CD166+ dual positive cells were typically more than 90% of live cells. CD133-/CD166- dual negative cells were typically less than 10%. CD44 was used to determine CD44- and CD44+ cells among the dual positive or negative cells. 80~95% of live cells were CD44-/CD133+/CD166+ cells, 1~3% of live cells were CD44+/CD133+/CD166+ cells, and less than 2% of live cells were CD44-/CD133-/CD166- cells.
Gating strategy	Using the FSC/SSC gating, debris was removed by gating on the main cell population. Positivity threshold for each sample was defined on the basis of unstained cells. Identical positivity threshold was applied to all samples and representative plots and gating are provided with every flow cytometry figure.

☒ Tick this box to confirm that a figure exemplifying the gating strategy is provided in the Supplementary Information.
