# Supplementary material for: Heterogeneity and dynamics of active Kras-induced dysplastic lineages from mouse corpus stomach
Source: Nat Commun. 2019 Dec 5;10:5549. doi: 10.1038/s41467-019-13479-6 (PMC6895174; doi:10.1038/s41467-019-13479-6)
Supplement: Supplementary file 6 — Description of Additional Supplementary Files [file 41467_2019_13479_MOESM6_ESM.pdf]

Title: Supplementary Movie 1
Description: Live imaging movies of organoid growth in 3D cultures

Title: Supplementary Movie 2
Description: Live imaging movies of organoid growth in 3D cultures

Title: Supplementary Data1
Description: A list of genes used for gene ontology analysis.
